# Paediatric contacts with the UK out-of-hours primary care service and contact outcomes: a regional service evaluation

**DOI:** 10.1186/s12875-020-01205-x

**Published:** 2020-07-14

**Authors:** George Edwards, Rachel Brettell, Chris Bird, Helen Hunt, Dan Lasserson, Gail Hayward

**Affiliations:** 1 Nuffield Department of Primary Care Health Sciences, Radcliffe Observatory Quarter, Woodstock Road, Oxford, OX2 6GG England; 2 Birmingham Children’s Hospital, Steelhouse Lane, Birmingham, B4 6NH England; 3 Oxford Health NHS Foundation Trust, Warneford Hospital, Warneford Lane, Oxford, OX3 7JX England; 4 Institute of Applied Health Research, College of Medical and Dental Sciences, Murray Learning Centre, University of Birmingham, Edgbaston, Birmingham, B15 2TT England

## Abstract

**Background:**

Demand on hospital emergency departments for paediatric problems is increasing. However, the volume and nature of paediatric health demands placed on other parts of the urgent care system have not been explored. This understanding is an important first step in developing and improving out-of-hospital care. We aimed to describe the volume, nature, and outcomes of paediatric contacts with out-of-hours general practice (OOH GP). We performed a retrospective service evaluation using data from 12 months of paediatric patient contacts with the Oxfordshire OOH GP service.

**Methods:**

A database of contacts with the Oxfordshire OOH GP service was created for a 12 month period from December 2014 to November 2015. Descriptive statistics were calculated using SPSS Version 25.

**Results:**

27,455 contacts were made by 18,987 individuals during a 12 month period. The majority of these were for children aged under 5. Over 70% of contacts were at the weekend. The peak contact period was between 18:30 and 21:30. Over 40% of contacts resulted in advice only (no onward referral, requirement for GP follow up, or prescription). 19.7% of contacts resulted in an antibiotic prescription, most commonly those linked with ear, chest, and throat infections.

**Discussion:**

Paediatric contacts with the Oxfordshire OOH GP service were predominantly in younger age groups and in the evening, with 19.7% resulting in an antibiotic prescription. Almost half of the contacts had no follow up or prescription, suggesting non-prescribing health care professionals could be involved in providing care in OOH GP. Further research should consider how children and their parents can be best supported to optimise OOH consulting.

## Background

Demand on hospital emergency departments for paediatric problems is increasing [1]^,^ [2]^,^ [3]. At one centre, attendances for medical problems in the Emergency Department (ED) rose 42% in the 10 years between 1997 and 8 and 2007–8 and the 0–4 years age group account for almost 70% of these contacts [3]. However, amongst children aged under 16, almost 30% of these contacts may be better managed in primary and out-of-hospital settings [4]^,^ [5]^,^ [6], including out-of-hours general practice (OOH GP).

In the UK, the provision of primary care services outside core contracted hours is an integral NHS service [7]^,^ [8]. In 2013–2014, 5.8 million cases were handled by OOH GP in England resulting in 3.3 million face-to-face consultations, including 800,000 home visits [9]. This service provides access to urgent primary care between 18:30 and 08:00 on weekdays, and all day on weekends and on bank holidays. It is an important alternative to a visit to the emergency department. Appointments with the OOH GP service are booked vie the free-to-use NHS 111 telephone advice line, where trained call handlers use the NHS pathways algorithm to direct patients to self-care advice or the most appropriate service for their needs.

Understanding the nature and outcomes of contacts with NHS services is key to evaluating and improving these services. Paediatric contacts with NHS services have been described in relation to Emergency Departments (ED) or Urgent Care Centres (UCC) [10–12] . In OOH primary care services in eight European countries, up to a third of contacts were shown to be for children under the age of 18 [13] and a third of out of hours consultations amongst under 12 s were fever related in a Dutch OOH GP service [14]. Of these, 7.6% were referred to secondary care [14]. In Denmark 27% of children aged 0–5 presenting to OOH GP received a prescription, 74% of which were for antibiotics, and 7.4% of children were referred to a nearby hospital [15].

To date, there has been no description of paediatric contacts, or the outcome of these contacts, with OOH GP in the UK. This understanding is critical to inform new approaches to managing the rising demand for acute paediatric assessment across urgent and unscheduled care, and ensuring safe and appropriate management. We aimed to characterise the nature, timing, and outcomes of paediatric contacts with the OOH GP using a large dataset of patient contacts with the Oxfordshire OOH service, which provides care to a population of over 600,000 people.

## Methods

A database of all intended patient contacts with the Oxfordshire OOH GP service, which provides care to a population of over 600,000 people, over 1 year from 1 December 2014 to 30 November 2015 was created from the OOH Electronic Record System used by clinicians (Adastra).

Service data included contact type, contact outcome, date, clinical codes assigned and prescriptions issued. Demographic data included patient sex, age, and a deprivation index (the Index of Multiple Deprivation (IMD)). Lower IMD scores indicate lower levels of deprivation. Contacts were coded with their final contact type: a ‘telephone consultation’, a ‘base visit’ (patient assessed at OOH base), a ‘home visit’ (patient assessed in their home), or a “111 appointment to book” for patients the 111 service have determined an appointment is needed but cannot be booked (for example due to appointments being full, or the triaging service lacking access to appointment system). This results in a contact with the OOH GP which may be a base visit, telephone consultation, or home visit.

Timings of calls were classified as evening 18:30–23:59 h, night 00:00–07:59 h, and daytime (on weekends and bank holidays) 08:00–18:29 h. The weekend period was classified as 18:30 h Friday until 08:00 h Monday, whilst contacts classified as ‘Bank Holiday’ were those occurring during daytime hours on each Bank Holiday.

At the end of each consultation, clinicians assign at least one clinical code (for example ‘H05z. Upper respiratory infection. NOS’), which were used in this study to determine clinical presentation for that contact. These clinical codes were validated by two members of the direct care team based on previous coding validity studies [16]. The positive predictive value (PPV) of the clinical code for medical diagnosis or conclusion was 92.6% [8]. In order to facilitate analysis these codes translated into International Classification of Primary Care (ICPC) codes by two clinically qualified authors (GH, RB) (supplementary Table 1). Codes which may indicate an infection were noted. An additional code, not available in the ICPC, was added for contacts indicating the patients did not attend (DNA). A small number of contacts (*n* = 264, 1%) had “NULL” as their clinical code. These contacts were removed from the dataset.

Clinicians also detail any prescriptions issued during the consultation in up to six fields. Prescriptions were grouped into 21 categories and 79 sub-categories for the purposes of analysis. These are detailed in supplementary Table 2.

Each contact is coded with an ‘Outcome’. There were 19 outcome codes, which were condensed into 9 outcome categories. These are detailed in supplementary Table 3.

Descriptive statistics were calculated in SPSS version 25. Analyses were undertaken at the contact level unless otherwise stated.

## Results

**Fig. 1:**
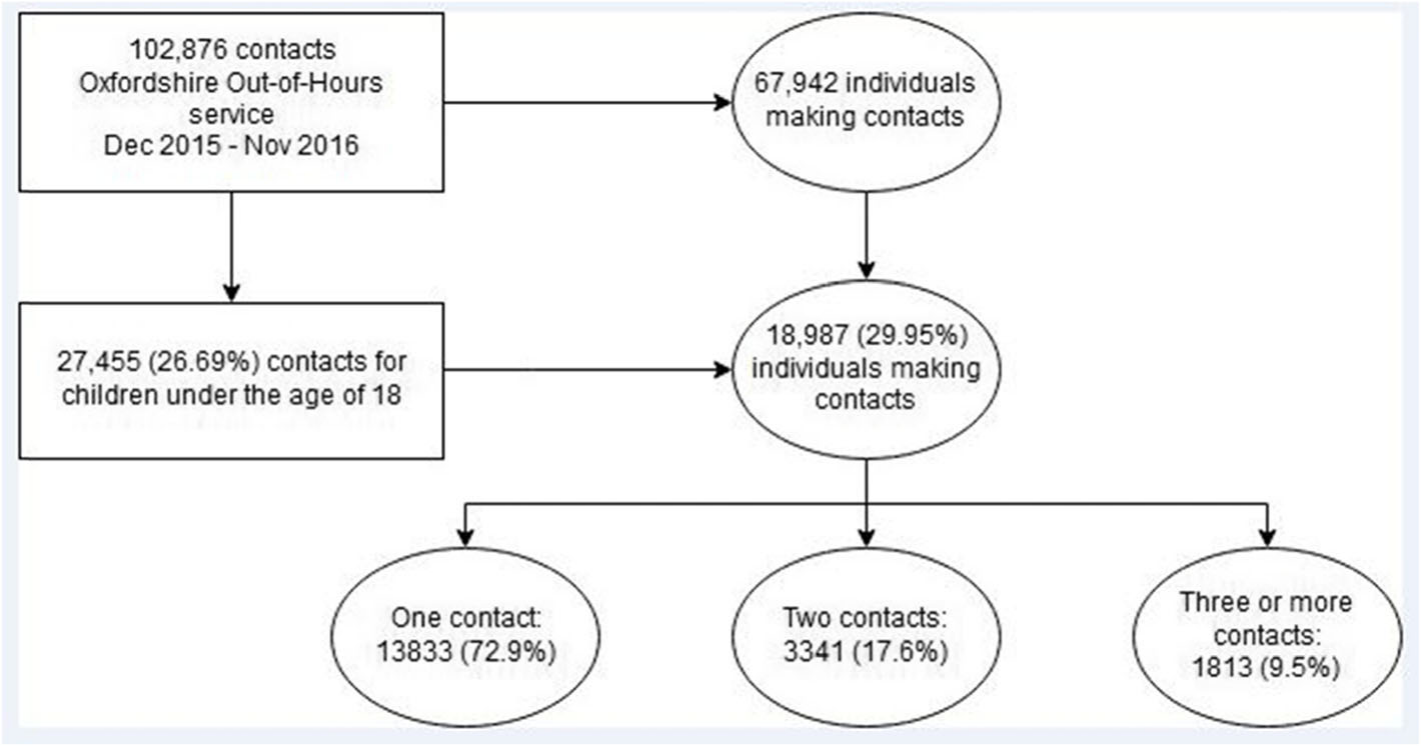
patient flow diagram

Between 1 December 2014 and 30 November 2015, 67,942 patients made 102,876 contacts with the Oxfordshire OOH GP service. Of these, 27,455 contacts (26.69%) made by 18,987 individuals (29.95%) were for children under the age of 18. Of these individuals, 13,833 (72.9%) had one contact, 3341 (17.6%) had two contacts, and 1813 (9.5%) had three or more contacts. See Fig. 1 for details.

The median age of children contacting the OOH GP service was 3.1 (IQR 1.1–7.0). In the twelve month period, 6136 contacts (22.3%) were for children under the age of one, and 11,852 (43.2%) of contacts were for children aged between 1 and 4 years. Children aged 5–11 account for 6118 contacts (22.3%), whilst there were 3349 contacts (12.2%) for children aged 12 and over. The median index of multiple deprivation (IMD) score was 10.79 (IQR 6.15–18.24).

**Fig. 2:**
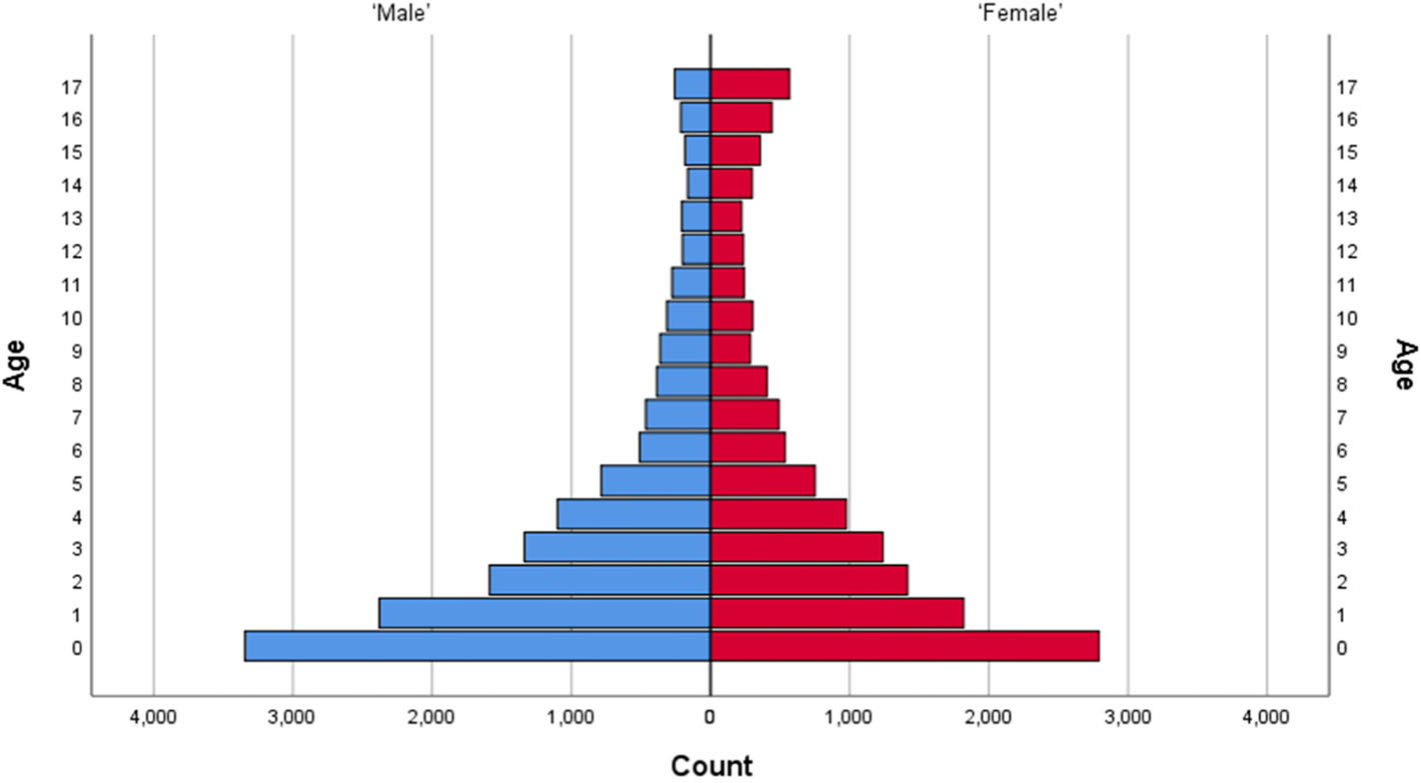
Population pyramid of frequency of contact age by sex seen by the Oxfordshire OOH service from December 2014 to November 2015

48.7% of contacts were for female patients, and of these the median age in years was 3.5 (IQR 1.2–8.1). The median age of male patients was 2.8 (IQR 1.1–6.0). Figure 2 shows the age distribution, in whole years, of all paediatric contacts with the Oxfordshire OOH GP service during the 12 month period. At younger ages, there were slightly more male patients (54.5 and 54.0% in the 0–1 and 1–5 age groups respectively). In contrast, in the 12–17 age group almost twice as many female patients contacted the service (63.5% of contacts in this age group).

### Contact types and timings

The month with the greatest number of contacts was December, and July had the lowest number of contacts (Table 1*).* 19,260 (70.2%) contacts were at the weekend and 870 (3.2%) were on a Bank Holiday. 9103 (33.2%) contacts were on a Saturday and 8048 (29.3%) were made on a Sunday. During the week, Tuesdays and Wednesdays had the lowest proportion of contacts (6.4% in both cases), followed by Thursday (7.0%), Monday (8.8%) and Friday (9.0%).

**Table 1:** The number and percentage of contacts in each month

Month	Number of contacts	Percent
December 2014	3522	12.8%
January 2015	2399	8.7%
February 2015	2407	8.8%
March 2015	2558	9.3%
April 2015	2307	8.4%
May 2015	2522	9.2%
June 2015	1819	6.6%
July 2015	1639	6.0%
August 2015	1668	6.1%
September 2015	1799	6.6%
October 2015	2136	7.8%
November 2015	2679	9.8%
Total	27,455	100.0%

There were 20,692 (75.4%) base visits whilst 6603 (24.1%) contacts were managed purely by telephone. There were 123 (0.4%) home visits and 37 (0.1%) “111 appt to book contacts” (contacts for which appointments cannot be booked within the time frame dictated by 111 and are passed onto the OOH GP service separately).

**Fig. 3:**
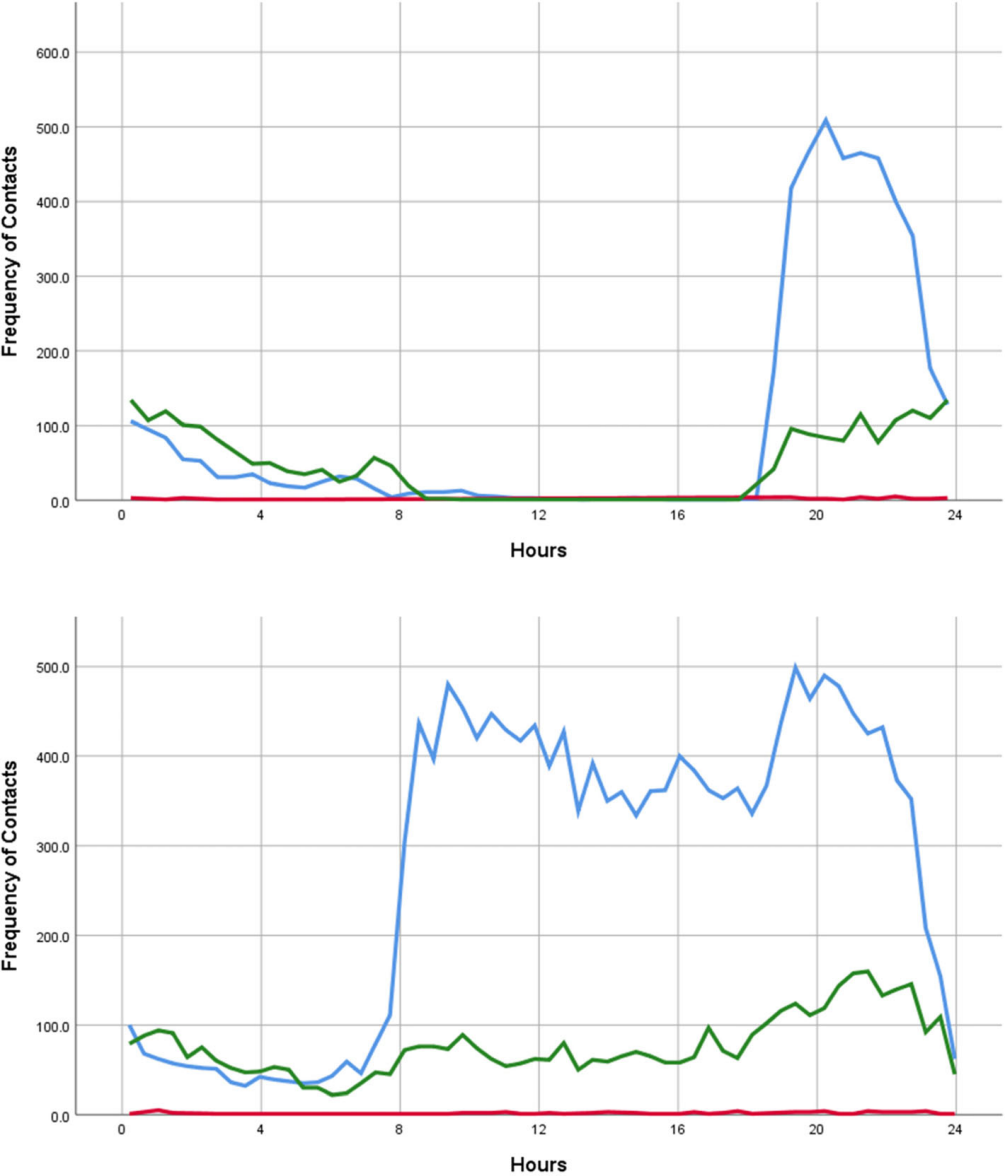
*Line graph by Final Contact Type showing the frequency of contacts by contact time with the OOH service through the day (top) during the week excluding Bank Holidays, and (bottom) at the weekend and on Bank Holidays (lower). Blue = “Base visit”, Green = “Telephone Consultation”, Red = “Home visit”*

Figure 3 demonstrates the pattern of attendance during weekdays (top) and at the weekend or on Bank Holidays (bottom) divided by type of contact (excluding “111 appt to book”). Peak attendance was between 18:00 and 21:00. On non-bank holiday weekdays, 5089 contacts (73.4%) were in the evening and 1749 (25.2%) contacts were in the night. In contrast at the weekend, 10779 (56.0%) contacts were made during the day, 2021 (10.5%) were during the night and 6457 (33.5%) were during the evening.

### Reasons for contact

Table 2 shows the frequency of contacts including at least one clinical code falling into each ICPC Chapter. The most common clinical category overall and in each age-category was ‘Respiratory’ (Chapter R), with related codes in 9280 (33.8%) contacts. Other commonly arising chapters ‘General and Unspecified’ (Chapter A) (*n* = 5627, 20.5%), ‘Digestive’ (Chapter D) (4181, 15.2%), ‘Skin’ (Chapter S) (2635, 9.6%), and ‘Ear’ (Chapter H) (2209, 8.0%). 472 (1.7%) of contacts were coded with a musculoskeletal code and this was most prevalent in children aged 12 and older. 82 (2.4%) contacts for children aged 12 and over were coded with a psychiatric condition. Ninety-four of the 101 of children aged less than 1 with a psychological code had problems feeding.

**Table 2:** The frequency and percentage of contacts with clinical codes falling into each ICPC Chapter

ICPC Chapter	Age Group									
	‘0’		‘1–4’		‘5–11’		‘12+’		Total No. Contacts with Clinical Category	
	Count	%	Count	%	Count	%	Count	%	Count	%
R - Respiratory	1942	31.6%	4650	39.2%	1917	31.3%	771	22.9%	9280	33.8%
A - General and unspecified	1603	26.1%	2864	24.2%	890	14.5%	270	8.1%	5627	20.5%
D - Digestive	1141	18.6%	1465	12.3%	995	16.2%	580	17.2%	4181	15.2%
S - Skin	478	7.8%	1055	8.9%	716	11.7%	386	11.5%	2635	9.6%
H - Ear	275	4.5%	1052	8.9%	684	11.1%	198	5.8%	2209	8.0%
Process	389	6.3%	521	4.4%	371	6.0%	299	8.8%	1580	5.6%
U - Urological	33	0.5%	303	2.6%	296	4.8%	228	6.8%	860	3.1%
D/F - DNA/Failed encounter^b^	178	2.9%	294	2.5%	122	2.0%	94	2.8%	688	2.5%
F - Eye	237	3.9%	276	2.3%	92	1.5%	62	1.9%	667	2.4%
L - Musculoskeletal	20	0.3%	95	0.8%	160	2.6%	197	5.9%	472	1.7%
N - Neurological	94	1.5%	89	0.8%	100	1.6%	139	4.2%	422	1.5%
Y - Male Genital	11	0.2%	124	1.0%	69	1.1%	23	0.7%	227	0.8%
P - Psychological	101	1.6%	10	0.1%	16	0.3%	82	2.4%	209	0.6%
X - Female Genital	11	0%	55	1%	33	1%	74	2%	173	0.6%
W - Pregnancy, Childbearing, Family Planning	1	0.0%	0	0.0%	0	0.0%	75	2.2%	76	0.3%
B - Blood, Blood Forming Organs and Immune Mechanism	6	0.1%	27	0.2%	18	0.3%	7	0.2%	58	0.2%
T - Endocrine/Metabolic and Nutritional	8	0.1%	18	0.2%	9	0.1%	17	0.5%	52	0.2%
K - Cardiovascular	3	0.0%	5	0.0%	11	0.2%	31	0.9%	50	0.2%
Z - Social Problems	0	0.0%	2	0.0%	2	0.0%	3	0.1%	7	0.0%
Total	6531	^*^	12,905	^*^	6501	^*^	3536	^*^	29,473	^*^

*Total percentages are not given. Some contacts have clinical code falling into more than one clinical category meaning the total percentages will be above 100%

^b^ Code created in addition to ICPC codes to reflect failed encounters and those where patients did not attend (DNA)

The most common ICPC Codes were Upper Respiratory Tract Infection (R74) (14.3% of contacts), Viral disease other/NOS (A77) (7.7%), Fever (A03) (7.1%), Tonsillitis Acute (R76) (4.9%), and Acute otitis media/myringitis (H71) (4.9%) (see supplementary Table 4 for full details). Across the data set, 14,267 (52.0%) individuals contacted the OOH GP service regarding an infection. Children aged 1–4 had the highest percentage of infection (59.6%).

### Outcomes of contacts

After 16,153 (58.8%) contacts there was no documented requirement for follow up. After 11,359 (41.4%) contacts there was no documented requirement for follow up and no prescription issued, considered as ‘advice only’ appointments. In 8394 (30.6%) of contacts the patient was advised to contact their own GP, and there was a referral to secondary care in 1754 (6.4%) of contacts. Table 3 shows the proportion of each contact outcome by age group.

**Table 3:** Frequency and percentage of contact outcomes by age group.

Contact Outcome	Age Group								Total No. Contacts	
	‘0’		‘1–4’		‘5–11’		‘12+’			
	Count	%	Count	%	Count	%	Count	%	Count	%
No Follow Up or Prescription	2941	47.9%	5049	42.6%	2282	37.3%	1087	32.5%	11,359	41.4%
No Follow Up with Prescription	671	10.9%	2122	17.9%	1319	21.6%	682	20.4%	4794	17.5%
Patient Advised to Contact Own GP	1782	29.0%	3548	29.9%	1943	31.8%	1121	33.5%	8394	30.6%
Referred to A&E	259	4.2%	449	3.8%	215	3.5%	168	5.0%	1091	4.0%
Admitted to Hospital	218	3.6%	246	2.1%	114	1.9%	85	2.5%	663	2.4%
Other referral	121	2.0%	181	1.5%	137	2.2%	120	3.6%	559	2.0%
No contact	101	1.6%	182	1.5%	75	1.2%	51	1.5%	409	1.5%
Own GP to contact patient	33	0.5%	49	0.4%	18	0.3%	30	0.9%	130	0.5%
Other	10	0.2%	26	0.2%	15	0.2%	5	0.1%	56	0.2%
Total	6136	100%	11,852	100%	6118	100%	3349	100%	27,455	100%

There were a total of 9633 prescriptions issued at 7840 (28.6%) contacts. In total, 5598 (29.5%) individual children received prescriptions. 5656 (58.7%) prescriptions were for antibiotics, and these were issued in 5420 (19.7%) of contacts. Of contacts resulting in antibiotic prescription, 2278 of these contacts (42.0%) had Respiratory codes (R), 1215 (22.4%) had Ear codes (H) and 778 (14.4%) had Skin codes (S) (Table 4).

**Table 4:** Count and % for each ICPC Chapter in contacts resulting in an antibiotic prescription

ICPC Chapter	Count	% Contacts receiving an antibiotic
R – Respiratory	2278	42.0%
H – Ear	1215	22.4%
S – Skin	778	14.4%
U – Urological	476	8.8%
A - General and unspecified	470	8.7%
F – Eye	330	6.1%
D – Digestive	129	2.4%
Process	121	2.2%
Y - Male Genital	98	1.8%
L – Musculoskeletal	25	0.0%
X - Female Genital	18	0.0%
B - Blood, Blood Forming Organs and Immune Mechanism	12	0.0%
N – Neurological	11	0.0%
K – Cardiovascular	2	0.0%
W - Pregnancy, Childbearing, Family Planning	2	0.0%
P – Psychological	1	0.0%
T - Endocrine/Metabolic and Nutritional	0	0.0%
Z - Social Problems	0	0.0%
D/F - DNA/Failed Encounter	0	0.0%

Prescriptions for asthma were the next most common with 852 (8.8%) prescriptions for inhaled asthma medication and devices issued in 567 (2.1%) contacts (Table 5). These included 529 (5.5%) prescriptions for bronchodilators, and 90 (0.9%) prescriptions for inhaled steroids. Steroid (oral and topical) prescriptions were also common with 679 (7.0%) prescriptions issued in 664 (2.4%) contacts.

**Table 5:** Count and % of prescribed items by category

Prescription Category	Count	%
Antibiotic	5656	58.7%
Inhaled Asthma Medication And Devices	852	8.8%
Steroid	679	7.0%
Analgesia	533	5.5%
Anti-Infective	405	4.2%
Allergy	357	3.7%
Topical Treatment	327	3.4%
Gastrointestinal Disease Medication	308	3.2%
Laxative	230	2.4%
Miscellaneous	137	1.4%
Contraception	39	0.4%
Antiemetic	33	0.3%
Psychiatric Medication	28	0.3%
Epilepsy	17	0.2%
Diabetes Care	10	0.1%
Diabetes	7	0.1%
Dressing	5	0.1%
Immunosuppression	4	0.0%
Formula	4	0.0%
Cardiac Medication	2	0.0%

## Discussion

### Summary of findings

Over a quarter (26.69%) of contacts with the Oxfordshire OOH GP service between December 2014 and November 2015 were for children under the age of 18. Of these, the majority (65.5%) were for children aged under 5 years. There were more male than female patients amongst the under 5 s, but amongst teenagers almost two thirds of the contacts were for female patients.

In over 40% of contacts there was no onward referral, requirement for GP follow up, or prescriptions, indicating that the primary clinical output of the contact was advice or reassurance. The proportion of these contacts was highest amongst the youngest age group (47.9%) and fell in each subsequent age group. Antibiotics were prescribed in 19.7% of contacts, most commonly linked with ear, respiratory, and skin conditions.

During the week, the majority of contacts were in the evening, with a peak between 18:30 and 21:30. At the weekend, there was an early peak of contacts at 09:00 and a second, higher peak between the hours of 19:00 and 21:00.

Children presented most commonly with respiratory conditions. The most common ICPC code was an upper respiratory tract infection. Other common codes were unspecified viral diseases, tonsillitis and acute otitis media. There were few contacts with musculoskeletal or psychological complaints.

### Comparison with the literature

In the UK research into paediatric use of NHS services has focused on unscheduled urgent care in Urgent Care Centres (UCCs) and the Emergency Department (ED). Use of health services is highest amongst the youngest children [10, 17]. In 2011–12, one in three children aged under five visited the ED compared with 17% of children aged 5–14 [10]. This use peaks at 18:00 on weekdays, although not at weekends [10]. Amongst children under 5 the most common complaints to a UCC are respiratory system, infectious, or parasitic diseases [11]. In contrast, older children, especially those aged over 15 [11], use primary care services less, but present more commonly to UCCs with musculoskeletal complaints [12]. Our findings suggest that the profile of paediatric demand on OOH GP is more similar to that of the ED than the UCC.

In Europe the proportion of paediatric contacts with OOH GP ranged from 1.9% in Switzerland to 33.3% in Denmark [13]. In Belgium, Denmark, the Netherlands, and Norway, contacts were highest for those in the 0–4 age group [13]. This work found that musculoskeletal and respiratory problems were very common amongst patients aged 0–17, as were ‘general and unspecified’ and ‘ear related’. Our study also found that respiratory and ear conditions were common but musculoskeletal conditions were less common. This may be due to the co-location of over half of the Oxfordshire bases with a minor injuries unit which would typically assess musculoskeletal problems. Similarly, urgent patient problems related to psychological illnesses may be handled by parallel out-of-hours services. These differences may be interesting to explore further in the context of the remit of other OOH GP available in these countries.

There is limited research into the outcomes of paediatric consultations with OOH GP. One study of consultations for fever amongst children consulting with an OOH GP service in the Netherlands found that 92% of contacts were managed without referral to secondary care [14]. In our study the equivalent figure was 93.6%.

Antibiotics were prescribed in 19.7% of contacts in this study, primarily for respiratory, ear, and skin conditions. Antibiotic prescribing has previously been associated with 15% of all contacts with the UK OOH GP service, and 18.0% of contacts for individuals under the age of 18 [18]. Although our data were from the same population, it was from a later time period; reasons for a higher antibiotic prescription rate are unclear. Qualitative work has revealed that GPs have a lower threshold for prescribing OOH [19]. They raised concerns including safety, a lack of background knowledge about patients in an OOH GP setting and a lack of availability of diagnostics [19].

### Strength and limitations

To our knowledge this is the first study to explore in detail paediatric demand on a UK OOH GP service. It uses a large dataset of 27,455 contacts. However, as the dataset is limited to Oxfordshire, our results may not be generalisable to the UK population as a whole. Oxfordshire is, on the whole, less deprived than the UK, and our methods should be replicated in other regions with different populations and care models. These data also precede recent extended access schemes which may alter how people navigate the health services.

### Implications for research and practice

We found that 41.4% of contacts were ‘advice only’. Whilst these contacts may have been appropriate, this highlights the need for research exploring the content and main function of these consultations, and reviewing whether alternative sources of advice could offer a safe substitute. This also suggests that nurse practitioners and paramedics who do not prescribe could be part of the team offering paediatric assessment with appropriate training.

Antibiotics were prescribed in 19.7% of contacts. UK OOH GP is associated with a disproportionately high rate of antibiotic prescribing [20], representing 5% of prescriptions from primary care despite only 1% of GP consultations occurring out-of-hours. In the Netherlands the appropriateness of antibiotic prescribing in OOH GP is comparable to in-hours care [21], but the extent to which this is true in the UK is unclear and could be a focus of future research. Point of care CRP testing has been shown to be feasible in a UK OOH primary care service [22], however, its role in reducing prescribing for acute respiratory illness in paediatrics remains unclear in this setting [23].

Finally, our study took a clinician perspective on contacts with OOH GP linking the reason for contact with the clinical codes applied during the consultation. Qualitative research exploring the process by which parents make decisions to consult OOH GP specifically will deepen our understanding of these consultations and how patients can be best supported. This is of specific interest for general and respiratory illnesses amongst younger children.

## Conclusions

We found that paediatric contacts with the Oxfordshire OOH GP service were predominantly in younger age groups and in the early evening with 19.7% resulting in an antibiotic prescription. Almost half of the contacts were advice-only appointments, and further research should consider how patients making these appointments can be best supported.

## Supplementary information

**Additional file 1.**

**Additional file 2: Supplementary Table 2.** Prescription Categorisation

**Additional file 3: Supplementary Table 3.** Outcome codes from the dataset and their reported category.

**Additional file 4.**

## Data Availability

No data available. Data sharing permissions are not in place.

## Abbreviations

OOH GP: Out-of-hours general practice
ED: Emergency department
IQR: Inter quartile range
UCC: Urgent care centre
ICPC: International classification of primary care
NOS: Not otherwise specified
IMD: Index of Multiple Deprivation
