# Supplementary material for: Paediatric contacts with the UK out-of-hours primary care service and contact outcomes: a regional service evaluation
Source: BMC Fam Pract. 2020 Jul 14;21:144. doi: 10.1186/s12875-020-01205-x (PMC7362454; doi:10.1186/s12875-020-01205-x)
Supplement: Supplementary file 1 — Additional file 1. [file 12875_2020_1205_MOESM1_ESM.docx]

| Adastra Code | ICPC Chapter | ICPC Code | ICPC Name | Notes |
| --- | --- | --- | --- | --- |
| ZV6.. [V]Other reasons for encounter | - | -69 | Other reason for encounter NEC |  |
| 8H... Referral-other | - | -68 | Other referral NEC |  |
| 8HH.. Referred - other care | - | -66 | Referral to non-MD provider |  |
| 8Hkh. Safeguarding children referral | - | -66 | Referral to non-MD provider |  |
| 892.. Informed consent for procedure | - | -62 | Administrative procedure |  |
| 9Z... Administration NOS | - | -62 | Administrative procedure |  |
| 419.. Lab. test result abnormal | - | -61 | Result examination/test/record/letter from other provider |  |
| 44TJ. Blood glucose level | - | -60 | Result test/procedure |  |
| 8B1.. Emergency treatment | - | -59 | Other therap proced/minor surg NEC |  |
| 9871. Minor surgery done + claimable | - | -59 | Other therap proced/minor surg NEC |  |
| 8.... Other Therapeutic Procedures | - | -59 | Other therap proced/minor surg NEC |  |
| 9k1A. Alcohol brief intervention | - | -58 | Therapeutic counselling/listening |  |
| 81H.. Dressing of wound | - | -56 | Dressing/pressure/compress/tamponade |  |
| 81H5. Change of dressing | - | -56 | Dressing/pressure/compress/tamponade |  |
| 8C1L. Wound care | - | -56 | Dressing/pressure/compress/tamponade |  |
| 7G2E. Dressing of skin | - | -56 | Dressing/pressure/compress/tamponade |  |
| 7G251 Drainage of abscess NEC | - | -51 | I&d/flush/aspiration |  |
| 8B3H. Medication requested | - | -50 | Medication/prescr/renewal |  |
| 8B3S. Medication review | - | -50 | Medication/prescr/renewal |  |
| 8B3z. Drug therapy NOS | - | -50 | Medication/prescr/renewal |  |
| 8B41. Repeated prescription | - | -50 | Medication/prescr/renewal |  |
| 8BAA. Pain Relief | - | -50 | Medication/prescr/renewal |  |
| 8B2.. Therapeutic prescription | - | -50 | Medication/prescr/renewal |  |
| 8BC2. Medication given | - | -50 | Medication/prescr/renewal |  |
| 6Z... Preventive procedures NOS | - | -49 | Other preventive procedure |  |
| 67E.. Foreign travel advice | - | -45 | Observ/health educat/advice/diet |  |
| 677B. Advice about treatment given | - | -45 | Observ/health educat/advice/diet |  |
| 67I.. Advice | - | -45 | Observ/health educat/advice/diet |  |
| 8CAK. Patient given telephone advice out of hours | - | -45 | Observ/health educat/advice/diet |  |
| 8CA.. Patient given advice | - | -45 | Observ/health educat/advice/diet |  |
| 8C9.. Reassurance given | - | -45 | Observ/health educat/advice/diet |  |
| 7L1g. Administration of vaccine | - | -44 | Preventive immunization/medic |  |
| 7P1Az Diagnostic blood tests NOS | - | -43 | Other diagnostic procedure |  |
| 235.. O/E - respiratory rate | - | -43 | Other diagnostic procedure |  |
| 321Z. ECG - general - NOS | - | -42 | Electrical tracing |  |
| 461.. MSU - general | - | -35 | Urine test |  |
| 41D0. Blood sample taken | - | -34 | Blood test |  |
| 9Oq0. Chlamydia test offered | - | -33 | Microbiological/immunological test |  |
| 2315. Chest examination | - | -31 | Medical examin/health eval partial |  |
| 38C1. Mental health assessment | - | -30 | Medical examin/health eval complete |  |
| ZV202 [V]Routine child health check | - | -30 | Medical examin/health eval complete |  |
| 2516. Abdominal examination | - | -30 | Medical examin/health eval complete |  |
| 8I3T. Chlamydia screening declined | - | -62 | Administrative procedure | Manually split into male and female |
| 1M... Pain | - | -62 | Administrative procedure |  |
| 1DC8. Generalised pain [symptom] | A | A01 | Pain general/multiple sites |  |
| R00z2 [D]Pain | A | A01 | Pain general/multiple sites |  |
| R0062 [D]Fever NOS | A | A03 | Fever |  |
| R006z (D) Pyrexia of unknown origin NOS | A | A03 | Fever |  |
| febrile | A | A03 | Fever |  |
| R0000 [D]Drowsiness | A | A04 | Weakness/tiredness general |  |
| R0070 [D]Malaise | A | A05 | Feeling ill |  |
| R007z (D) Malaise and Fatigue NOS | A | A05 | Feeling ill |  |
| R093. [D]Swelling | A | A08 | Swelling |  |
| R023. [D]Oedema | A | A08 | Swelling |  |
| 1828. Atypical chest pain | A | A11 | Chest pain NOS |  |
| R065B [D]Non cardiac chest pain | A | A11 | Chest pain NOS |  |
| 1B1I0 Crying infant | A | A16 | Irritable infant |  |
| Rz... Symptoms Signs and ill defined conditions NOS | A | A29 | General symptom/complaint other |  |
| 2236. O/E - unconscious/comatose | A | A29 | General symptom/complaint other |  |
| A55.. Measles | A | A71 | Measles |  |
| A52.. Chickenpox - varicella | A | A72 | Chickenpox |  |
| A84.. Malaria | A | A73 | Malaria |  |
| A56.. Rubella | A | A74 | Rubella |  |
| A75.. Infectious mononucleosis | A | A75 | Infectious mononucleosis |  |
| A5... Viral diseases with exanthem | A | A76 | Viral exanthem other |  |
| A79z. Viral infection NOS | A | A77 | Viral disease other/NOS |  |
| Az... Infectious and Parasitic Disease NOS | A | A78 | Infectious disease other/NOS |  |
| 65PZ. Communicable disease contact NOS | A | A78 | Infectious disease other/NOS |  |
| SP25. Postoperative infection | A | A78 | Infectious disease other/NOS |  |
| A0... Intestinal infectious diseases | A | A78 | Infectious disease other/NOS |  |
| A3C.. Sepsis | a | a78 | Infectious disease other/NOS |  |
| A38.. SEPTICAEMIA | a | a78 | Infectious disease other/NOS |  |
| TC... Accidental falls | A | A80 | Trauma/injury NOS |  |
| 16D.. Falls | A | A80 | Trauma/injury NOS |  |
| T1... Motor vehic.traffic acc.(MVTA) | A | A80 | Trauma/injury NOS |  |
| Sz... Injury and Poisoning NOS | A | A80 | Trauma/injury NOS |  |
| TE6.. Other injury caused by animals | A | A80 | Trauma/injury NOS |  |
| TE6z. Injury caused by animal NOS | A | A80 | Trauma/injury NOS |  |
| SL... Poisoning | A | A84 | Poisoning by medical agent |  |
| SN52. Drug hypersensitivity NOS | A | A85 | Adverse effect medical agent |  |
| TJ... Adverse Reaction to Drug | A | A85 | Adverse effect medical agent |  |
| T8... Accidental poisoning by drugs | A | A85 | Adverse effect medical agent |  |
| SN501 Anaphylactic shock due to adverse effect of correct drug or mediction properly administered | A | A85 | Adverse effect medical agent |  |
| SM3.. Corrosives - toxic effect | A | A86 | Toxic effect non-medicinal substance |  |
| SP2z. Postoperative complicat. NOS | A | A87 | Complication of medical treatment |  |
| SN16. Hypothermia - accidental | A | A88 | Adverse effect physical factor |  |
| SN41. Any drowning injury | A | A88 | Adverse effect physical factor |  |
| 9Ee0K Pathology report | A | A91 | Abnormal result investigation NOS |  |
| SN53. Allergy | A | A92 | Allergy/allergic reaction NOS |  |
| SN58. Food allergy | A | A92 | Allergy/allergic reaction NOS |  |
| SN590 Allergic reaction to bee sting | A | A92 | Allergy/allergic reaction NOS |  |
| SN582 Peanut allergy | A | A92 | Allergy/allergic reaction NOS |  |
| SN50. Anaphylactic shock | A | A92 | Allergy/allergic reaction NOS |  |
| SN51. Angioneurotic oedema | A | A92 | Allergy/allergic reaction NOS |  |
| 94Z.. Death Administration NOS | A | A96 | Death |  |
| 212A. O/E patient well | A | A97 | No disease |  |
| 2125. Nil abnormal on examination | A | A97 | No disease |  |
| 222G. Only use if diagnosis not made | A | A97 | No disease |  |
| 1692. Swollen glands | B | B02 | Lymph gland(s) enlarged/painful |  |
| M04.. Acute lymphadenitis | B | B70 | Lymphadenitis acute |  |
| D00.. Iron deficiency anaemias | B | B80 | Iron deficiency anaemia |  |
| D010. Other deficiency anaemias | B | B82 | Anaemia other/unspecified |  |
| 9N4C. Failed encounter - no answer when rang back | D/F | D/F |  |  |
| 9N4.. Failed encounter | D/F | D/F |  |  |
| 9N4G. Failed Encounter - phone number unobtainable | D/F | D/F |  |  |
| 9N47. Cancelled | D/F | D/F |  |  |
| 9N41. Did not attend - reason given | D/F | D/F |  |  |
| 9Ni.. Did not attend | D/F | D/F |  |  |
| 9N42. Did not attend -no reason | D/F | D/F |  |  |
| 9N45. Patient walked out | D/F | D/F |  |  |
| 8I4.. Patient self discharge | D/F | D/F |  |  |
| R090. [D]Abdominal pain | D | D01 | Abdominal pain/cramps general |  |
| J574F Anorectal pain | D | D01 | Abdominal pain/cramps general |  |
| J521. Irritable Colon (IBS) | D | D01 | Abdominal pain/cramps general |  |
| R0903 [D]Infantile colic | D | D01 | Abdominal pain/cramps general |  |
| R080. [D]Renal colic | D | D06 | Abdominal pain localized other |  |
| J16y4 Dyspepsia | D | D07 | Dyspepsia/indigestion |  |
| R0700 [D]Nausea | D | D09 | Nausea |  |
| R0701 [D]Vomiting | D | D10 | Vomiting |  |
| 19F2. Diarrhoea | D | D11 | Diarrhoea |  |
| A0820 Dysenteric diarrhoea | D | D11 | Diarrhoea |  |
| J520. Constipation | D | D12 | Constipation |  |
| 19C2. Constipated | D | D12 | Constipation |  |
| J66y6 Obstructive Jaundice NOS | D | D13 | Jaundice |  |
| J680. Haematemesis | D | D14 | Haematemesis/vomiting blood |  |
| J681. Melaena | D | D15 | Melaena |  |
| J573. Haemorrhage of rectum and anus | D | D16 | Rectal bleeding |  |
| 19E6. Blood in faeces? -if altered blood or Melaena use code J681. Melaena | D | D16 | Rectal bleeding |  |
| J007. Teething syndrome | D | D19 | Teeth/gum symptom/complaint |  |
| 1912. Toothache | D | D19 | Teeth/gum symptom/complaint |  |
| S8360 Open wound in mouth | D | D19 | Teeth/gum symptom/complaint |  |
| S8363 Broken tooth injury | D | D19 | Teeth/gum symptom/complaint |  |
| 19... Gastrointestinal symptoms | D | D29 | Digestive symptom/complaint other |  |
| 7N324 [SO]Colorectal | D | D29 | Digestive symptom/complaint other |  |
| J68.. Gastrointestinal haemorrhage | D | D29 | Digestive symptom/complaint other |  |
| J6z.. Liver/biliary/pancreas+GIT NOS | D | D29 | Digestive symptom/complaint other |  |
| J571. Rectal prolapse | D | D29 | Digestive symptom/complaint other |  |
| A07y0 Viral gastroenteritis | D | D70 | Gastrointestinal infection |  |
| A72.. Mumps | D | D71 | Mumps |  |
| A0812 Gastroenteritis - presumed inf | D | D73 | Gastroenteritis presumed infection |  |
| S7... Any other internal or visceral injury | D | D80 | Injury digestive system other |  |
| J0z.. Oral/salivary/jaw diseases NOS | D | D83 | Mouth/tongue/lip disease |  |
| J05.. Other dental diseases and conditions of the teeth and supporting structures | D | D83 | Mouth/tongue/lip disease |  |
| J1011 Reflux Oesophagitis | D | D84 | Oesophagus disease |  |
| J15.. Gastritis or Duodenitis | D | D87 | Stomach function disorder |  |
| J20.. Acute appendicitis | D | D88 | Appendicitis |  |
| J30.. Inguinal Hernia | D | D89 | Inguinal hernia |  |
| J3... Hernia of abdominal cavity | D | D91 | Abdominal hernia other |  |
| J4... Non infective enteritis | D | D94 | Chronic enteritis/ulcerative colitis |  |
| J53.. Anal Fissure and Fistula | D | D95 | Anal fissure/perianal abscess |  |
| J54.. Abscess of Anal/Rectal Regions | D | D95 | Anal fissure/perianal abscess |  |
| Jz... Digestive System Diseases NOS | D | D99 | Disease digestive system other |  |
| J17.. Other stomach/duodenal disord. | D | D99 | Disease digestive system other |  |
| J5... Other diseases of intestines and peritoneum. | D | D99 | Disease digestive system other |  |
| J670. Acute pancreatitis | D | D99 | Disease digestive system other |  |
| F48.. Visual disturbances | F | F05 | Visual disturbance other |  |
| 1B72. Diplopia/double vision | F | F05 | Visual disturbance other |  |
| 7N133 [SO]Eyelid | F | F16 | Eyelid symptom/complaint |  |
| F4... Disorders of eye and adnexa | F | F29 | Eye symptom/complaint other |  |
| F4C0. Acute Conjunctivitis | F | F70 | Conjunctivitis infectious |  |
| F4C06 Acute atopic conjunctivitis | F | F70 | Conjunctivitis infectious |  |
| F4005 Eye infection | F | F73 | Eye infection/inflammation other |  |
| F4G01 Orbital cellulitis | F | F73 | Eye infection/inflammation other |  |
| F4C03 Acute mucopur. conjunctivitis | F | F73 | Eye infection/inflammation other |  |
| F4K0. Episcleritis | F | F73 | Eye infection/inflammation other |  |
| F4C71 Subconjunctival haemorrhage | F | F75 | Contusion/haemorrhage eye |  |
| SG0.. Foreign body (FB) on external eye | F | F76 | Foreign body in eye |  |
| SD8.. Superficial injury of eye and adnexa | F | F79 | Injury eye other |  |
| 1C3.. Earache symptoms | H | H01 | Ear pain/earache |  |
| F583. Tinnitus | H | H03 | Tinnitus, ringing/buzzing ear |  |
| 1C42. Ear discharge present | H | H04 | Ear discharge |  |
| 1C... Ear symptoms | H | H29 | Ear symptom/complaint other |  |
| 1C8.. Nasal symptoms OS | H | H29 | Ear symptom/complaint other |  |
| 1C84. C/O - post nasal drip | H | H29 | Ear symptom/complaint other |  |
| F56.. Vestibular syndromes/disorders | H | H29 | Ear symptom/complaint other |  |
| F502z Otitis externa NOS | H | H70 | Otitis externa |  |
| F52z. Otitis media NOS | H | H71 | Acute otitis media/myringitis |  |
| F5120 Glue ear | H | H72 | Serous otitis media |  |
| F51y0 Eustachian tube dysfunction | H | H73 | Eustachian salpingitis |  |
| SG1.. Foreign body in ear | H | H76 | Foreign body in ear |  |
| F504. Impacted Cerumen (Wax In Ear) | H | H81 | Excessive ear wax |  |
| F5611 Benign paroxysm.posit.vertigo | H | H82 | Vertiginous syndrome |  |
| F59z. Deafness NOS | H | H86 | Deafness |  |
| F5z.. Ear and Mastoid Disease NOS | H | H99 | Ear/mastoid disease other |  |
| F58z. Ear disorder NOS | H | H99 | Ear/mastoid disease other |  |
| R065. [D]Chest pain | K | K01 | Heart pain |  |
| 1812. Palpitations | K | K04 | Palpitations/awareness of heart |  |
| R050. [D]Tachycardia | K | K29 | Cardiovascular sympt/complt other |  |
| G57.. Cardiac dysrhythmias | K | K80 | Cardiac arrhythmia NOS |  |
| R0521 [D]Heart murmur | K | K81 | Heart/arterial murmur NOS |  |
| G84.. Haemorrhoids | K | K96 | Haemorrhoids |  |
| G.... Circulatory system diseases | K | K99 | Cardiovascular disease other |  |
| N13.. Neck Disorder Inc. Torticollis | L | L01 | Neck symptom/complaint |  |
| N131. Cervicalgia - pain in neck | L | L01 | Neck symptom/complaint |  |
| N145. Backache | L | L02 | Back symptom/complaint |  |
| N142. Pain in lumbar spine | L | L02 | Back symptom/complaint |  |
| N141. Pain in thoracic spine | L | L02 | Back symptom/complaint |  |
| N2457 Shoulder pain | L | L08 | Shoulder symptom/complaint |  |
| N21.. Shoulder Syndromes | L | L08 | Shoulder symptom/complaint |  |
| 1M00. Pain in elbow | L | L10 | Elbow symptom/complaint |  |
| N094K Arthralgia of hip | L | L13 | Hip symptom/complaint |  |
| 1M10. Knee pain | L | L15 | Knee symptom/complaint |  |
| N07.. Internal derangement of knee | L | L15 | Knee symptom/complaint |  |
| N216. Knee Syndromes/Bursitis/Tendinitis | L | L15 | Knee symptom/complaint |  |
| S5yz1 Muscle injury / strain | L | L19 | Muscle symptom/complaint NOS |  |
| N094. Pain in joint - arthralgia | L | L20 | Joint symptom/complaint NOS |  |
| N095. Joint stiffness NEC | L | L20 | Joint symptom/complaint NOS |  |
| S4... Dislocations and subluxations | L | L20 | Joint symptom/complaint NOS |  |
| N245. Pain in limb | L | L29 | Musculoskeletal sympt/complt other |  |
| N096. Other joint symptoms | L | L29 | Musculoskeletal sympt/complt other |  |
| S2... Fracture of upper limb | L | L75 | Fracture: femur |  |
| S020. Fracture of nose | L | L76 | Fracture: other |  |
| S1... Fracture of any spinal vertebrae or rib fracture | L | L76 | Fracture: other |  |
| S3zz. Fracture Of Bones NOS | L | L76 | Fracture: other |  |
| S20.. Fracture of clavicle | L | L76 | Fracture: other |  |
| S3z00 Greenstick fracture | L | L76 | Fracture: other |  |
| sprains and strains involving thorax with lower back and pelvis | L | L79 | Sprain/strain of joint NOS |  |
| sprains and strains involving head with neck | L | L79 | Sprain/strain of joint NOS |  |
| S5z.. Sprains and strains NOS | L | L79 | Sprain/strain of joint NOS |  |
| S44z. Dislocation of finger or thumb not otherwise specified | L | L80 | Dislocation/subluxation |  |
| SR20. Dislocations | L | L80 | Dislocation/subluxation |  |
| SR21. Dislocations | L | L80 | Dislocation/subluxation |  |
| SD5.. Superficial of injury finger(s) | L | L81 | Injury musculoskeletal NOS |  |
| SD7.. Superficial injury of foot and toe(s) | L | L81 | Injury musculoskeletal NOS |  |
| SD... Superficial injury | L | L81 | Injury musculoskeletal NOS |  |
| SD3.. Superficial injury of elbow | L | L81 | Injury musculoskeletal NOS |  |
| SDz.. Superficial injuries NOS | L | L81 | Injury musculoskeletal NOS |  |
| N143. Sciatica | L | L86 | Back syndrome with radiating pain |  |
| N223. Bursitis | L | L87 | Bursitis/tendinitis/synovitis NOS |  |
| 1JG.. Suspected inflammatory arthritis | L | L88 | Rheumatoid/seropositive arthritis |  |
| N040. Rheumatoid Arthritis | L | L88 | Rheumatoid/seropositive arthritis |  |
| Nz... Musculoskeletal diseases NOS | L | L99 | Musculoskeletal disease other |  |
| N0... Arthropathies and related dis. | L | L99 | Musculoskeletal disease other |  |
| N224. Ganglion and synovial cyst | L | L99 | Musculoskeletal disease other |  |
| 1BA2. Generalised headache | N | N01 | Headache |  |
| 1B1G. Headache | N | N01 | Headache |  |
| R0206 [D] Numbness | N | N06 | Sensation disturbance other |  |
| R003z [D]Convulsion NOS | N | N07 | Convulsion/seizure |  |
| R0030 [D]Convulsions | N | N07 | Convulsion/seizure |  |
| R004. [D]Dizziness and giddiness | N | N17 | Vertigo/dizziness |  |
| R002z [D]Syncope and collapse NOS | N | N29 | Neurological sympt/complt other |  |
| R0023 [D]Collapse | N | N29 | Neurological sympt/complt other |  |
| F132z Myoclonus NOS | N | N29 | Neurological sympt/complt other |  |
| R0161 [D] Meningism NOS | N | N29 | Neurological sympt/complt other |  |
| 2239. O/E - decreased level of consciousness | N | N29 | Neurological sympt/complt other |  |
| 2234. O/E - drowsy | N | N29 | Neurological sympt/complt other |  |
| R009. [D] Confusion | N | N29 | Neurological sympt/complt other |  |
| SD0.. Superficial injury to head | N | N80 | Head injury other |  |
| S646. Head injury | N | N80 | Head injury other |  |
| F25.. Epilepsy | N | N88 | Epilepsy |  |
| F26.. Migraine | N | N89 | Migraine |  |
| F310. Bell's (facial) palsy | N | N91 | Facial paralysis/bell's palsy |  |
| F2626 [X] Tension type headache | N | N95 | Tension headache |  |
| Fz... Nervous system or sense organ disease NOS | N | N99 | Neurological disease other |  |
| F02.. Meningitis - unspecified cause | N | N99 | Neurological disease other |  |
| 1JA.. Suspected neurological disease | N | N99 | Neurological disease other |  |
| None | None | None | None |  |
| Unspecified | None | None | None |  |
| unspecif | None | None | None |  |
| forearm and wrist | None | None | None |  |
| no infec | None | None | None |  |
| mass | None | None | None |  |
| excluding fingers | None | None | None |  |
| palm | None | None | None |  |
| undiagnosed | None | None | None |  |
| site NOS | None | None | None |  |
| excluding eye | None | None | None |  |
| organism unsp | None | None | None |  |
| 423.. Haemoglobin estimation | None | None | None |  |
| heel | None | None | None |  |
| generalized | None | None | None |  |
| childbirth and the purepurium NOS | None | None | None |  |
| lump abd/pelv | None | None | None |  |
| Non-Organic | None | None | None |  |
| 1B13. Anxiousness | P | P01 | Feeling anxious/nervous/tense |  |
| E28.. Acute Reaction To Stress/Panic Attacks | P | P02 | Acute stress reaction |  |
| 1B17. Depressed | P | P03 | Feeling depressed |  |
| R0050 [D]Sleep disturbance | P | P06 | Sleep disturbance |  |
| R0330 [D]Feeding problem in infant | P | P11 | Eating problem in child |  |
| Eu102 [X]Alcohol dependence syndrome | P | P15 | Chronic alcohol abuse |  |
| E.... Mental disorders | P | P29 | Psychological sympt/compl other |  |
| U2z.. Intentional Self Harm by unspecified means | P | P29 | Psychological sympt/compl other |  |
| R00zD [D]Restlessness and agitation | P | P29 | Psychological sympt/compl other |  |
| R00zW [D]State emotion shock+stress | P | P29 | Psychological sympt/compl other |  |
| 1B19. Suicidal | P | P29 | Psychological sympt/compl other |  |
| TK0.. Suicide + selfinflicted poisoning | P | P29 | Psychological sympt/compl other |  |
| 2233. O/E - delirious | p | p71 | Organic psychosis other |  |
| E030z Acute confusional state NOS | P | P71 | Organic psychosis other |  |
| E1... Schizophrenia | P | P72 | Schizophrenia |  |
| E200. Anxiety States | P | P74 | Anxiety disorder/anxiety state |  |
| E2B.. Depressive disorder NEC | P | P76 | Depressive disorder |  |
| E21.. Personality disorders | P | P80 | Personality disorder |  |
| Affect.Psychoses | P | P98 | Psychosis NOS/other |  |
| Ez... Mental Disorders NOS | P | P99 | Psychological disorders other |  |
| 1825. Pleuritic pain | R | R01 | Pain respiratory system |  |
| R0653 [D]Painful respiration NOS | R | R01 | Pain respiratory system |  |
| 173.. Breathlessness | R | R02 | Shortness of breath/dyspnoea |  |
| 1739. Shortness of breath | R | R02 | Shortness of breath/dyspnoea |  |
| 1737. Wheezing | R | R03 | Wheezing |  |
| 173e. Viral wheeze | R | R03 | Wheezing |  |
| 232C. Noisy breathing | R | R04 | Breathing problem other |  |
| 1738. Difficulty breathing | R | R04 | Breathing problem other |  |
| R061. [D]Stridor | R | R04 | Breathing problem other |  |
| R0601 Hyperventilation | R | R04 | Breathing problem other |  |
| R062. [D]Cough | R | R05 | Cough |  |
| R047. [D]Epistaxis | R | R06 | Nose bleed/epistaxis |  |
| 1CBZ. Throat symptom NOS | R | R21 | Throat symptom/complaint |  |
| 1C93. Persistent sore throat | R | R21 | Throat symptom/complaint |  |
| 1C92. Has a sore throat | R | R21 | Throat symptom/complaint |  |
| 1CB.. Throat symptom NOS | R | R21 | Throat symptom/complaint |  |
| 1CB5. Throat irritation | R | R21 | Throat symptom/complaint |  |
| 1CB3. Throat pain | R | R21 | Throat symptom/complaint |  |
| R063. [D]Haemoptysis | R | R24 | Haemoptysis |  |
| 1731. No breathlessness | R | R29 | Respiratory symptom/complaint other |  |
| H02.. Acute pharyngitis | R | R74 | Upper respiratory infection acute |  |
| H05z. Upper respiratory infect.NOS | R | R74 | Upper respiratory infection acute |  |
| H01.. Acute sinusitis | R | R75 | Sinusitis acute/chronic |  |
| H03.. Acute Tonsillitis | R | R76 | Tonsillitis acute |  |
| H044. Croup | R | R77 | Laryngitis/tracheitis acute |  |
| H04.. Acute laryngitis/tracheitis | R | R77 | Laryngitis/tracheitis acute |  |
| H06.. Acute bronchitis/bronchiolitis | R | R78 | Acute bronchitis/bronchiolitis |  |
| H061. Acute bronchiolitis | R | R78 | Acute bronchitis/bronchiolitis |  |
| 16L.. Influenza-like symptoms | R | R80 | Influenza |  |
| H27z. Flu-like illness | r | r80 | Influenza |  |
| 1J72. Suspected influenza A virus subtype H1N1 infection | r | r80 | Influenza |  |
| H21.. Lobar (pneumococcal) pneumonia | R | R81 | Pneumonia |  |
| H22.. Other bacterial pneumonia | R | R81 | Pneumonia |  |
| H25.. Bronchopneumonia | R | R81 | Pneumonia |  |
| H20.. Viral pneumonia | R | R81 | Pneumonia |  |
| H51.. Pleurisy | R | R82 | Pleurisy/pleural effusion |  |
| H06z1 Lower resp tract infection | R | R83 | Respiratory infection other |  |
| H11.. Nasal polyps | R | R86 | Benign neoplasm respiratory |  |
| 663V0 Occasional asthma | R | R96 | Asthma |  |
| 663V1 Mild asthma | R | R96 | Asthma |  |
| 663V2 Moderate asthma | R | R96 | Asthma |  |
| H33.. Asthma | R | R96 | Asthma |  |
| H333. Acute exacerbation of asthma | R | R96 | Asthma |  |
| H33z. Asthma unspecified | R | R96 | Asthma |  |
| H17.. Allergic rhinitis | R | R97 | Allergic rhinitis |  |
| H120. Chronic rhinitis | R | R99 | Respiratory disease other |  |
| H5... Other resp.system diseases | R | R99 | Respiratory disease other |  |
| Hz... Respiratory System Diseases NOS | R | R99 | Respiratory disease other |  |
| M18z. Pruritus NOS | S | S02 | Pruritus |  |
| N24.. Any soft tissue inflammation | S | S05 | Lumps/swellings generalized |  |
| R021z [D] Rash/nonspec. skin eruption | S | S07 | Rash generalized |  |
| TE640 Insect bite NOS | S | S12 | Insect bite/sting |  |
| U127. [X]Bit/stung - nonvenom insect | S | S12 | Insect bite/sting |  |
| SD65. Insect bite nonv.-leg+infectn. | S | S12 | Insect bite/sting |  |
| SD64. Insect bite nonv.-leg | S | S12 | Insect bite/sting |  |
| SN591 Allergic reaction to insect bite | S | S12 | Insect bite/sting |  |
| TE60. Dog bite | S | S13 | Animal/human bite |  |
| TE6y8 Scratched by cat | S | S13 | Animal/human bite |  |
| 8I5.. Care/help refused by patient | S | S13 | Animal/human bite |  |
| TE630 Cat bite | S | S13 | Animal/human bite |  |
| TLxy0 Assault by bite of human being | S | S13 | Animal/human bite |  |
| SH... Burns | S | S14 | Burn/scald |  |
| 7G245 Removal of foreign body from skin NEC | S | S15 | Foreign body in skin |  |
| SE... Contusion (bruise)+intact skin | S | S16 | Bruise/contusion |  |
| SE4z. Contusion | S | S16 | Bruise/contusion |  |
| S8z.. Laceration | S | S18 | Laceration/cut |  |
| S930. Open wound of finger or thumb without mention of complication | S | S19 | Skin injury other |  |
| SA... Open wound of lower limb | S | S19 | Skin injury other |  |
| S9200 Open wound of hand | S | S19 | Skin injury other |  |
| SA3.. Open wound of toe(s) | S | S19 | Skin injury other |  |
| S8... Open wound of head/neck trunk | S | S19 | Skin injury other |  |
| S9... Open wound of upper limb | S | S19 | Skin injury other |  |
| SA203 Open wound | S | S19 | Skin injury other |  |
| S834. Open wound of face | S | S19 | Skin injury other |  |
| S8362 Open wound of gum | S | S19 | Skin injury other |  |
| S910. Open wound of lower arm without mention of complication | S | S19 | Skin injury other |  |
| SD4.. Superficial injury of hand | S | S19 | Skin injury other |  |
| SF... Crushing injury | S | S19 | Skin injury other |  |
| SD1.. Superficial injury of trunk | S | S19 | Skin injury other |  |
| 7G321 Avulsion of Nail | S | S22 | Nail symptom/complaint |  |
| SE330 Subungual haematoma | S | S22 | Nail symptom/complaint |  |
| M1271 Sunburn | S | S29 | Skin symptom/complaint other |  |
| 7N7.. [SO]Skin | S | S29 | Skin symptom/complaint other |  |
| A53.. Herpes zoster | S | S70 | Herpes zoster |  |
| A54.. Herpes Simplex | S | S71 | Herpes simplex |  |
| AD30. Scabies | S | S72 | Scabies/other acariasis |  |
| AB... Mycoses | S | S74 | Dermatophytosis |  |
| M163. Pityriasis rosea | S | S74 | Dermatophytosis |  |
| M0... Skin/subcutaneous infections | S | S76 | Skin infection other |  |
| M03z0 Cellulitis NOS | S | S76 | Skin infection other |  |
| M06.. Pilonidal sinus/cyst | S | S85 | Pilonidal cyst/fistula |  |
| M11.. Atopic dermatitis and related | S | S87 | Dermatitis/atopic eczema |  |
| M111. Atopic dermatitis/eczema | S | S87 | Dermatitis/atopic eczema |  |
| M1100 Candidal nappy rash | S | S89 | Diaper rash |  |
| M110. Napkin dermatitis | S | S89 | Diaper rash |  |
| M16.. Psoriasis and similar disord. | S | S91 | Psoriasis |  |
| M230. Ingrowing Nail | S | S94 | Ingrowing nail |  |
| A780. Molluscum contagiosum | S | S95 | Molluscum contagiosum |  |
| M28.. Urticaria | S | S98 | Urticaria |  |
| Mz... Skin & Subcutaneous Disease NOS | S | S99 | Skin disease other |  |
| M2z.. Other skin/subcut.disease NOS | S | S99 | Skin disease other |  |
| M1... Other skin/subcut.inflam.dis. | S | S99 | Skin disease other |  |
| M26.. Sebaceous gland diseases | S | S99 | Skin disease other |  |
| D3121 Purpura | S | S99 | Skin disease other |  |
| 2225. O/E - dehydrated | T | T11 | Dehydration |  |
| C112. Hypoglycaemia unspecified | T | T87 | Hypoglycaemia |  |
| C108. Insulin depnd diabetes melitus | T | T89 | Diabetes insulin dependent |  |
| C10.. Diabetes mellitus | T | T90 | Diabetes non-insulin dependent |  |
| C.... Endocrine and metabolic | T | T99 | Endocr/metab/nutrit disease other |  |
| R081. [D] Dysuria | U | U01 | Dysuria/painful urination |  |
| 1A23. Incontinence of urine | U | U04 | Incontinence urine |  |
| K197. Haematuria | U | U06 | Haematuria |  |
| R082. [D] Retention of urine | U | U08 | Urinary retention |  |
| 7N51. [SO]Lower urinary tract | U | U29 | Urinary symptom/complaint other |  |
| K1652 Bladder outflow obstruction | U | U29 | Urinary symptom/complaint other |  |
| 4613. Urinalysis = no abnormality | U | U29 | Urinary symptom/complaint other |  |
| K101. Acute pyelonephritis | U | U70 | Pyelonephritis/pyelitis |  |
| K190. Urinary tract infection | U | U71 | Cystitis/urinary infection other |  |
| K15.. Cystitis | U | U71 | Cystitis/urinary infection other |  |
| K1905 Urinary Tract Infection | U | U71 | Cystitis/urinary infection other |  |
| K12.. Calculus of kidney and ureter | U | U95 | Urinary calculus |  |
| Kz... Genitourinary Disease NOS | U | U99 | Urinary disease other |  |
| K1... Other urinary system diseases | U | U99 | Urinary disease other |  |
| 61M.. Emergency contraception | W | W10 | Contraception postcoital |  |
| 61AZ. Post-coital contraception or advice | W | W10 | Contraception postcoital |  |
| 614.. Oral contraception | W | W11 | Contraception oral |  |
| 61... Contraception | W | W14 | Contraception female other |  |
| 8CAw. Advice about long acting reversible contraception | W | W14 | Contraception female other |  |
| L1... Pregnancy complications | W | W29 | Pregnancy symptom/complaint other |  |
| L100. Threatened Abortion | W | W29 | Pregnancy symptom/complaint other |  |
| Lz... Complications of pregnancy | W | W29 | Pregnancy symptom/complaint other |  |
| 62... Patient pregnant | W | W78 | Pregnancy |  |
| L03.. Ectopic pregnancy | W | W80 | Ectopic pregnancy |  |
| L04.. Spontaneous Abortion | W | W82 | Abortion spontaneous |  |
| L14.. Early or threatened labour | W | W92 | Compl labour/ delivery livebirth |  |
| 1A581 Vulval pain | X | X01 | Genital pain female |  |
| R090G [D]Pelvic and perineal pain | X | X01 | Genital pain female |  |
| K583. Dysmenorrhoea | X | X02 | Menstrual pain |  |
| K5920 Menorrhagia | X | X06 | Menstruation excessive |  |
| K596. Metrorrhagia | X | X08 | Intermenstrual bleeding |  |
| K59yx Dysfunctional uterine haemorrhage NOS | X | X08 | Intermenstrual bleeding |  |
| K3108 Breast infection | X | X21 | Breast sympt/compl female other |  |
| K58.. Female genital organ symptoms | X | X29 | Genital sympt/compl female other |  |
| AB2y4 Anogenital candidosis | X | X72 | Genital candidiasis female |  |
| AB21. Candidal vulvovaginitis | X | X72 | Genital candidiasis female |  |
| K421. Vaginitis and vulvovaginitis | X | X84 | Vaginitis/vulvitis NOS |  |
| K5... Other female genital tract dis | X | X99 | Genital disease female other |  |
| 7C242 Standard circumcision | Y | Y04 | Penis symptom/complaint other |  |
| A9871 Gonococcal rectal infection | Y | Y71 | Gonorrhoea male |  |
| K24.. Orchitis and epididymitis | Y | Y74 | Orchitis/epididymitis |  |
| K242. Epididymo-orchitis | Y | Y74 | Orchitis/epididymitis |  |
| K241. Epididymitis | Y | Y74 | Orchitis/epididymitis |  |
| K271. Balanoposthitis | Y | Y75 | Balanitis |  |
| K23.. Hydrocele | Y | Y86 | Hydrocoele |  |
| K2... Male genital organ diseases | Y | Y99 | Genital disease male other |  |
| K27.. Disorders of Penis | Y | Y99 | Genital disease male other |  |
| K28.. Other male genital organ dis. | Y | Y99 | Genital disease male other |  |
| U3L.. [X]Sex assault by bodily force | Z | Z25 | Assault/harmful event problem |  |
| 14X8. Victim of domestic violence | Z | Z25 | Assault/harmful event problem |  |
| U3... [X]Assault | Z | Z25 | Assault/harmful event problem |  |
| 64c.. Child protection procedure | Z | Z29 | Social problem NOS |  |
| K59.. Menstruation disorders | X |  | Could be: X02 X05 X06 X07 | Could be: X02 X05 X06 X07 |
| 67AZ. Pregnancy advice NOS |  |  | Could be: -45 W78 | Could be: -45 W78 - both used in data |
| S3... Fracture of lower limb | L |  | Could be: L73 L74 L75 | Could be: L73 L74 L75 |
| A995. Nonspecific genital infection |  |  |  | Manually split into male and female (X99, Y99) |
| A541. Genital herpes simplex |  |  |  | Manually split into male and female (X90 Y72) |
| A5417 Genital herpes simplex type 2 |  |  |  | Manually split into male and female (X90 Y72) |
| A7... Other viral/chlamydial diseas. |  |  |  | Manually split into male and female (X92 Y99) |
| A78A1 Chlamydia Pharyngeal infection |  |  |  | Manually split into male and female (X92 Y99) |
| K3... Disorders of breast | X |  | Could be: X18 X19 X20 X21 X22 | Could be: X18 X19 X20 X21 X22 |
| SG... Foreign body (FB) in orifice |  |  | Could be: D79 H76 R87 -59 | Could be: D79 H76 R87 -59 - all used in data |
