# Supplementary material for: Paediatric contacts with the UK out-of-hours primary care service and contact outcomes: a regional service evaluation
Source: BMC Fam Pract. 2020 Jul 14;21:144. doi: 10.1186/s12875-020-01205-x (PMC7362454; doi:10.1186/s12875-020-01205-x)
Supplement: Supplementary file 2 — Additional file 2: Supplementary Table 2. Prescription Categorisation [file 12875_2020_1205_MOESM2_ESM.docx]

**Supplementary Table 2: Prescription Categorisation**

| Name | Category | SubCategory |
| --- | --- | --- |
| Abidec Multivitamin | Otc | Multivitamins |
| Acetic Acid | Topical Treatment | Ear Spray |
| Aciclovir | Anti-Infective | Antiviral |
| Adrenaline | Allergy | Adrenaline |
| Altacite plus | Gastrointestinal Disease Medication | Antacid |
| Amoxicillin | Antibiotic | Antibiotic |
| Anugesic-Hc cream | Steroid | Topical Steroid |
| Aqueous Cream | Topical Treatment | Emollient |
| Aripiprazole | Psychiatric Medication | Antipsychotic |
| Aspirin | Analgesia | Nsaid |
| Atrovent | Inhaled Asthma Medication And Devices | Bronchodilator |
| Aviva test strips | Diabetes Care | Bm Testing Strip |
| Azathioprine | Immunosuppresion | Immunosuppressant |
| Azelastine | Allergy | Antihistamine |
| Azithromycin | Antibiotic | Antibiotic |
| Bactroban | Antibiotic | Antibiotic |
| Balneum | Topical Treatment | Emollient |
| Beclometasone | Inhaled Asthma Medication And Devices | Asthma Preventer |
| beclomethasone diproprionate | Inhaled Asthma Medication And Devices | Asthma Preventer |
| Beconase Nasal Spray | Steroid | Nasal Steroid |
| Benzydamine | Analgesia | Nsaid |
| Betametasone and Neomycin | Steroid | Steroid Drops |
| Betamethasone | Steroid | Topical Steroid |
| Bisacodyl | Laxative | Laxative |
| Bonjela | Topical Treatment | Topical Anti-Inflammatory |
| Calamine Lotion | Topical Treatment | Topical Cream |
| Capasal | Miscellaneous | Therapeutic Shampoo |
| Carbamazepine | Epilepsy | Anticonvulsant |
| Cavilon | Topical Treatment | Topical Cream |
| Cefaclor | Antibiotic | Antibiotic |
| Cefalexin | Antibiotic | Antibiotic |
| Cetirizine | Allergy | Antihistamine |
| Cetraben Cream | Topical Treatment | Emollient |
| Cetrimide Cream | Anti-Infective | Antiseptic |
| Chloramphenicol | Antibiotic | Antibiotic |
| Chlorhexidine | Anti-Infective | Antiseptic |
| Chloromycetin | Antibiotic | Antibiotic |
| Chlorphenamine | Allergy | Antihistamine |
| Choline | Topical Treatment | Topical Anti-Inflammatory |
| Ciprofloxacin | Antibiotic | Antibiotic |
| Clarithromycin | Antibiotic | Antibiotic |
| Clindamycin | Antibiotic | Antibiotic |
| Clobavate | Steroid | Topical Steroid |
| Clobazam | Psychiatric Medication | Benzodiazepine |
| Clobetasol | Steroid | Topical Steroid |
| Clobetasone | Steroid | Topical Steroid |
| Clotrimazole | Anti-Infective | Antifungal |
| Clotrimazole + hydrocortizone | Anti-Infective | Antifungal |
| Co-Amoxiclav | Antibiotic | Antibiotic |
| Co-Codamol | Analgesia | Weak Opiate |
| Codeine | Analgesia | Weak Opiate |
| Conotrane | Topical Treatment | Topical Cream |
| Cyclizine | Antiemetic | Antiemetic |
| Dermol Cream | Topical Treatment | Emollient |
| Dermol Lotion | Topical Treatment | Emollient |
| Dermovate | Steroid | Topical Steroid |
| Desloratadine | Allergy | Antihistamine |
| Dexamethasone | Steroid | Steroid |
| Diazepam | Psychiatric Medication | Benzodiazepine |
| Diclofenac | Analgesia | Nsaid |
| Dicycloverine | Gastrointestinal Disease Medication | Antispasmodic |
| Difflam Rinse | Topical Treatment | Topical Analgesic |
| Difflam Spray | Topical Treatment | Topical Analgesic |
| Diprobase Cream | Topical Treatment | Emollient |
| Docusate | Laxative | Laxative |
| Domperidone | Antiemetic | Antiemetic |
| Doublebase Gel | Topical Treatment | Emollient |
| Doxycycline | Antibiotic | Antibiotic |
| Drapolene | Topical Treatment | Barrier Cream |
| E45 Cream | Topical Treatment | Emollient |
| Econazole Cream | Anti-Infective | Antifungal |
| Elocon Ointment | Steroid | Topical Steroid |
| Emedastine | Allergy | Antihistamine |
| Emerade | Allergy | Adrenaline |
| Emla Cream | Topical Treatment | Topical Anaesthetic |
| Emulsiderm Emollient | Topical Treatment | Emollient |
| Emulsifying Ointment | Topical Treatment | Emollient |
| Epaderm Cream | Topical Treatment | Emollient |
| Epaderm Ointment | Topical Treatment | Emollient |
| Ephedrine | Psychiatric Medication | Stimulant |
| Epipen | Allergy | Adrenaline |
| erythromycin | Antibiotic | Antibiotic |
| Ethinylestradiol | Contraception | Contraception |
| Eucerin Lotion | Topical Treatment | Emollient |
| Eumovate Cream | Steroid | Topical Steroid |
| Eurax Cream | Topical Treatment | Emollient |
| Fastclix Lancets | Diabetes Care | Bm Lancet |
| Ferrous fumarate | Miscellaneous | Iron |
| Ferrous gluconate | Miscellaneous | Iron |
| Fexofenadine | Miscellaneous | Iron |
| Fifty:50 | Topical Treatment | Emollient |
| Flixonase | Steroid | Topical Steroid |
| Flucloxacillin | Antibiotic | Antibiotic |
| Fluconazole | Anti-Infective | Antifungal |
| Fludrocortisone | Steroid | Steroid |
| flumetasone and clioquinol drops | Steroid | Topical Steroid |
| Fluoxetine | Psychiatric Medication | Ssri |
| Freestyle Optium Testing Strips | Diabetes Care | Bm Testing Strip |
| Fucibet Cream | Antibiotic | Antibiotic |
| Fucidin Cream | Antibiotic | Antibiotic |
| Fucidin H cream | Antibiotic | Antibiotic |
| Fucithalmic Eye Drops | Antibiotic | Antibiotic |
| Fusidic Acid Cream | Antibiotic | Antibiotic |
| Fusidic acid drop | Antibiotic | Antibiotic |
| Fybogel | Laxative | Laxative |
| Gaviscon | Gastrointestinal Disease Medication | Antacid |
| Gaviscon Infant | Gastrointestinal Disease Medication | Antacid |
| Gentamicin | Antibiotic | Antibiotic |
| Gentisone H | Antibiotic | Antibiotic |
| GlucoRx needles | Diabetes Care | Needles |
| Glycerol | Laxative | Laxative |
| GTN ointment | Topical Treatment | Topical Gtn |
| Haloperidol | Psychiatric Medication | Antipsychotic |
| Hydrocortisone | Steroid | Topical Steroid |
| Hydromol Ointment | Topical Treatment | Emollient |
| Hydroxyzine | Allergy | Antihistamine |
| Hyoscine butylbromide | Gastrointestinal Disease Medication | Antispasmodic |
| Hypromellose | Topical Treatment | Eye Lubricant |
| Ibuprofen | Analgesia | Nsaid |
| Infacol | Otc | Colic Remedy |
| Instillagel | Topical Treatment | Topical Anaesthetic |
| Ipratropium | Inhaled Asthma Medication And Devices | Asthma Reliever |
| K-Y Jelly | Otc | Lubricant |
| Lactulose | Laxative | Laxative |
| Lamotrigine | Epilepsy | Antiepileptic |
| Lansoprazole | Gastrointestinal Disease Medication | Ppi |
| Lantus | Diabetes | Insulin |
| Laxido Orange | Laxative | Laxative |
| Levemir | Diabetes | Insulin |
| Levetiracetam | Epilepsy | Antiepileptic |
| Levocetirizine | Allergy | Antihistamine |
| Levofloxacin | Antibiotic | Antibiotic |
| Levonelle | Contraception | Emergency Contraception |
| Levonorgestrel | Contraception | Emergency Contraception |
| Levothyroxine | Miscellaneous | Thyroid Replacement |
| Lidocaine | Miscellaneous | Anaesthetic |
| Locorten-Voiform | Steroid | Topical Steroid |
| Loestrin | Contraception | Contraception |
| Loperamide | Gastrointestinal Disease Medication | Antidiarrhoeal |
| Loratadine | Allergy | Antihistamine |
| Lotriderm | Steroid | Topical Steroid |
| Lymecycline | Antibiotic | Antibiotic |
| magnesium glycerophosphate | Miscellaneous | Magnesium Replacement |
| magnesium sulfate paste | Miscellaneous | Magnesium Replacement |
| Malarone | Anti-Infective | Antimalarial |
| Maxitrol | Steroid | Topical Steroid |
| Mebendazole | Anti-Infective | Anthelmintic |
| mebeverine | Gastrointestinal Disease Medication | Antispasmodic |
| Mefenamic Acid | Analgesia | Nsaid |
| Melatonin | Psychiatric Medication | Melatonin |
| Menthol | Topical Treatment | Emollient |
| Metanium | Topical Treatment | Topical Cream |
| Methotrexate | Immunosuppresion | Dmard |
| methylphenidate | Psychiatric Medication | Stimulant |
| Metoclopramide | Antiemetic | Antiemetic |
| Metronidazole | Antibiotic | Antibiotic |
| miconazole | Steroid | Topical Steroid |
| miconazole and hydrocortizone | Steroid | Topical Steroid |
| Miconazole Cream | Anti-Infective | Antifungal |
| Miconazole Gel | Anti-Infective | Antifungal |
| Micralax | Laxative | Laxative |
| Micropore Tape | Dressing | Dressing |
| Midazolam | Psychiatric Medication | Benzodiazepine |
| Migraleve | Analgesia | Anti-Migraine |
| Mometasone | Steroid | Topical Steroid |
| Montelukast | Miscellaneous | Leukotriene Receptor Antagonist |
| Morphine | Analgesia | Strong Opiate |
| Movicol | Laxative | Laxative |
| Mucodyne | Inhaled Asthma Medication And Devices | Mucolytic |
| Mupirocin | Antibiotic | Antibiotic |
| Naproxen | Analgesia | Nsaid |
| Naseptin Nasal Cream | Antibiotic | Antibiotic |
| Nelsons Burns Cream | Topical Treatment | Topical Cream |
| Neocate | Formula | Infant Formula |
| Nitrofurantoin | Antibiotic | Antibiotic |
| No- atrovent is the inhaler, ipratropium is the drug (and here it is via nebuliser rather than via and inhaler_ | Inhaled Asthma Medication And Devices | Bronchodilator |
| Norethisterone | Contraception | Contraception |
| Novofine needles | Diabetes Care | Needles |
| Novorapid | Diabetes | Insulin |
| Nutramigen | Formula | Infant Formula |
| Nystatin | Anti-Infective | Antifungal |
| Oilatum | Topical Treatment | Emollient |
| Olopatadine | Allergy | Antihistamine |
| Omeprazole | Gastrointestinal Disease Medication | Ppi |
| Optichamber spacer | Inhaled Asthma Medication And Devices | Spacer |
| Optilast | Allergy | Anti-Allergic |
| oral rehydration salts | Otc | Oral Rehydration |
| Oraldene | Otc | Mouthwash |
| Otex | Topical Treatment | Ear Spray |
| Otomize Ear Spray | Steroid | Topical Steroid |
| Otosporin | Antibiotic | Antibiotic |
| Oxytetracycline | Antibiotic | Antibiotic |
| Paracetamol | Analgesia | Paracetamol |
| Paramax | Analgesia | Paracetamol |
| Peak Flow Meter | Inhaled Asthma Medication And Devices | Peak Flow Meter |
| Penicillin | Antibiotic | Antibiotic |
| Pentasa | Immunosuppresion | Aminosalicylate |
| Permethrin Cream | Anti-Infective | Insecticide |
| Phenergan | Allergy | Antihistamine |
| Piroxicam | Analgesia | Nsaid |
| Prednisolone | Steroid | Steroid |
| Prochlorperazine | Antiemetic | Antiemetic |
| Proctosedyl | Steroid | Topical Steroid |
| Proguanil | Anti-Infective | Antimalarial |
| Promethazine | Allergy | Antihistamine |
| Propranolol | Cardiac Medication | Betablocker |
| Pseudoephedrine | Otc | Decongestant |
| Quetiapine | Psychiatric Medication | Antipsychotic |
| Ranitidine | Gastrointestinal Disease Medication | Antacid |
| Rigevidon | Contraception | Contraception |
| Rufinamide | Epilepsy | Anticonvulsant |
| Salactol | Otc | Wart Treatment |
| Salbutamol | Inhaled Asthma Medication And Devices | Bronchodilator |
| Saline for nebuliser | Miscellaneous | Saline Neb |
| Seretide | Inhaled Asthma Medication And Devices | Asthma Preventer |
| Sertraline | Psychiatric Medication | Antidepressant |
| Simeticone | Otc | Anti-Colic |
| Simple | Otc | Linctus |
| Sodium alginate/magnesium alginate | Miscellaneous | Multiple |
| Sodium Bicarbonate | Miscellaneous | Multiple |
| Sodium Chloride | Miscellaneous | Multiple |
| Sodium cromoglicate | Allergy | Anti-Allergic |
| Sodium Picosulfate | Miscellaneous | Multiple |
| Sodium Valporate | Miscellaneous | Multiple |
| sodium valproate | Epilepsy | Antiepileptic |
| Sofradex Ear/Eye Drops | Antibiotic | Antibiotic |
| Softpore Dressing | Dressing | Dressing |
| Spacer | Inhaled Asthma Medication And Devices | Spacer |
| Sterimar | Otc | Decongestant |
| Strefen | Otc | Throat Lozenge |
| Sumatriptan | Analgesia | Triptan |
| Symbicort | Inhaled Asthma Medication And Devices | Asthma Preventer |
| Tegretol | Epilepsy | Antiepileptic |
| Terbinafine Cream | Anti-Infective | Antifungal |
| Terbutaline | Inhaled Asthma Medication And Devices | Asthma Reliever |
| Timodine Cream | Anti-Infective | Antifungal |
| Topiramate | Epilepsy | Antiepileptic |
| Tramadol | Analgesia | Weak Opiate |
| Tranexamic acid | Miscellaneous | Antifibrinolytic |
| Trimethoprim | Antibiotic | Antibiotic |
| Trimovate Cream | Antibiotic | Antibiotic |
| Ulipristal | Contraception | Emergency Contraception |
| Verapamil | Cardiac Medication | Antiarrhythmic |
| Xylometazoline | Otc | Decongestant |
| Xyloproct Ointment | Steroid | Topical Steroid |
| Zinc and Castor Oil Ointment | Topical Treatment | Topical Cream |
| Zithromax | Antibiotic | Antibiotic |
| Zopiclone | Psychiatric Medication | Z Drug |
| Zovirax Ointment | Anti-Infective | Antiviral |
