# Supplementary material for: Paediatric contacts with the UK out-of-hours primary care service and contact outcomes: a regional service evaluation
Source: BMC Fam Pract. 2020 Jul 14;21:144. doi: 10.1186/s12875-020-01205-x (PMC7362454; doi:10.1186/s12875-020-01205-x)
Supplement: Supplementary file 3 — Additional file 3: Supplementary Table 3. Outcome codes from the dataset and their reported category. [file 12875_2020_1205_MOESM3_ESM.docx]

# Supplementary Tables

| Outcome Code | Outcome Category |
| --- | --- |
| Admitted to hospital | Admitted to hospital |
| Unable to contact patient | Unable to contact patient |
| Did not attend | Unable to contact patient |
| Left before treatment | Unable to contact patient |
| No Follow Up | No Follow Up |
| (Blank) | Other |
| PGD Used | Other |
| Passed To Another Provider Patient Advised To Contact Own GP | Other |
| Passed To Another Provider | Other Referral |
| Minor Injuries Unit | Other Referral |
| Outpatient clinic | Other Referral |
| Referred To Community Nursing/Midwife | Other Referral |
| Mental Health Team | Other Referral |
| Social Services | Other Referral |
| EMU | Other Referral |
| Hospice Referral | Other Referral |
| Own GP to contact patient | Own GP to contact patient |
| Patient Advised To Contact Own GP | Patient Advised To Contact Own GP |
| Referred To A&E | Referred To A&E |

*Supplementary Table 3 – Outcome codes from the dataset and their reported category.*
