# Supplementary material for: Paediatric contacts with the UK out-of-hours primary care service and contact outcomes: a regional service evaluation
Source: BMC Fam Pract. 2020 Jul 14;21:144. doi: 10.1186/s12875-020-01205-x (PMC7362454; doi:10.1186/s12875-020-01205-x)
Supplement: Supplementary file 4 — Additional file 4. [file 12875_2020_1205_MOESM4_ESM.docx]

|  | AgeCat | ‘0' | ‘1-4’ | ‘5-11’ | ‘12+’ | Total |
| --- | --- | --- | --- | --- | --- | --- |
| ICPC Code | ICPC Code Name | Count | Count | Count | Count | Count |
| R74 | Upper respiratory infection acute | 1100 | 2005 | 652 | 179 | 3936 |
| A77 | Viral disease other/NOS | 555 | 1102 | 366 | 78 | 2101 |
| A03 | Fever | 518 | 1117 | 266 | 43 | 1944 |
| H71 | Acute otitis media/myringitis | 180 | 724 | 375 | 70 | 1349 |
| R76 | Tonsillitis acute | 60 | 667 | 430 | 188 | 1345 |
| D01 | Abdominal pain/cramps general | 121 | 215 | 444 | 274 | 1054 |
| D70 | Gastrointestinal infection | 266 | 500 | 191 | 61 | 1018 |
| R05 | Cough | 248 | 487 | 157 | 57 | 949 |
| S07 | Rash generalized | 192 | 395 | 162 | 50 | 799 |
| D10 | Vomiting | 291 | 296 | 104 | 55 | 746 |
| -45 | Observ/health educat/advice/diet | 237 | 277 | 141 | 80 | 735 |
| U71 | Cystitis/urinary infection other | 29 | 230 | 242 | 190 | 691 |
| D/F | Did not attend / Failed Encounter | 178 | 294 | 122 | 94 | 688 |
| R83 | Respiratory infection other | 113 | 403 | 116 | 56 | 688 |
| H01 | Ear pain/earache | 62 | 304 | 245 | 58 | 669 |
| S76 | Skin infection other | 62 | 240 | 232 | 135 | 669 |
| R21 | Throat symptom/complaint | 23 | 244 | 240 | 117 | 624 |
| R03 | Wheezing | 110 | 360 | 96 | 27 | 593 |
| R77 | Laryngitis/tracheitis acute | 69 | 402 | 72 | 5 | 548 |
| -50 | Medication/prescr/renewal | 56 | 107 | 119 | 125 | 407 |
| F70 | Conjunctivitis infectious | 165 | 179 | 30 | 14 | 388 |
| A76 | Viral exanthem other | 125 | 202 | 47 | 10 | 384 |
| S99 | Skin disease other | 98 | 148 | 100 | 38 | 384 |
| D12 | Constipation | 120 | 154 | 66 | 29 | 369 |
| R96 | Asthma | 2 | 106 | 176 | 85 | 369 |
| A72 | Chickenpox | 54 | 211 | 55 | 13 | 333 |
| D11 | Diarrhoea | 154 | 111 | 32 | 18 | 315 |
| R78 | Acute bronchitis/bronchiolitis | 192 | 87 | 10 | 5 | 294 |
| R04 | Breathing problem other | 79 | 158 | 40 | 10 | 287 |
| A16 | Irritable infant | 211 | 61 | 4 | 0 | 276 |
| D83 | Mouth/tongue/lip disease | 72 | 75 | 55 | 40 | 242 |
| S98 | Urticaria | 9 | 95 | 76 | 33 | 213 |
| D73 | Gastroenteritis presumed infection | 31 | 80 | 45 | 26 | 182 |
| A92 | Allergy/allergic reaction NOS | 17 | 59 | 64 | 27 | 167 |
| N80 | Head injury other | 82 | 48 | 17 | 17 | 164 |
| F29 | Eye symptom/complaint other | 34 | 48 | 44 | 29 | 155 |
| D19 | Teeth/gum symptom/complaint | 52 | 27 | 41 | 34 | 154 |
| H70 | Otitis externa | 6 | 28 | 65 | 52 | 151 |
| R99 | Respiratory disease other | 40 | 77 | 18 | 12 | 147 |
| S87 | Dermatitis/atopic eczema | 50 | 48 | 28 | 19 | 145 |
| -69 | Other reason for encounter NEC | 37 | 58 | 28 | 11 | 134 |
| -56 | Dressing/pressure/compress/tamponade | 13 | 27 | 36 | 50 | 126 |
| N01 | Headache | 1 | 14 | 51 | 55 | 121 |
| F73 | Eye infection/inflammation other | 43 | 47 | 13 | 15 | 118 |
| R80 | Influenza | 12 | 43 | 32 | 29 | 116 |
| A97 | No disease | 56 | 33 | 16 | 6 | 111 |
| Y75 | Balanitis | 3 | 72 | 33 | 2 | 110 |
| S89 | Diaper rash | 49 | 57 | 3 | 0 | 109 |
| Y99 | Genital disease male other | 6 | 51 | 31 | 15 | 103 |
| L29 | Musculoskeletal sympt/complt other | 6 | 25 | 39 | 29 | 99 |
| S12 | Insect bite/sting | 3 | 18 | 45 | 33 | 99 |
| P11 | Eating problem in child | 94 | 2 | 0 | 0 | 96 |
| -62 | Administrative procedure | 17 | 24 | 23 | 10 | 74 |
| A85 | Adverse effect medical agent | 9 | 30 | 18 | 15 | 72 |
| A78 | Infectious disease other/NOS | 12 | 32 | 15 | 9 | 68 |
| R06 | Nose bleed/epistaxis | 12 | 21 | 25 | 8 | 66 |
| H29 | Ear symptom/complaint other | 25 | 12 | 19 | 8 | 64 |
| A05 | Feeling ill | 23 | 28 | 5 | 7 | 63 |
| A80 | Trauma/injury NOS | 28 | 17 | 11 | 7 | 63 |
| X72 | Genital candidiasis female | 4 | 36 | 17 | 5 | 62 |
| L99 | Musculoskeletal disease other | 2 | 5 | 15 | 36 | 58 |
| L81 | Injury musculoskeletal NOS | 6 | 19 | 19 | 12 | 56 |
| D29 | Digestive symptom/complaint other | 20 | 15 | 10 | 9 | 54 |
| U99 | Urinary disease other | 0 | 29 | 20 | 4 | 53 |
| U01 | Dysuria/painful urination | 0 | 27 | 19 | 5 | 51 |
| D16 | Rectal bleeding | 20 | 14 | 8 | 8 | 50 |
| L02 | Back symptom/complaint | 0 | 3 | 10 | 37 | 50 |
| L20 | Joint symptom/complaint NOS | 1 | 10 | 19 | 20 | 50 |
| B02 | Lymph gland(s) enlarged/painful | 5 | 25 | 13 | 6 | 49 |
| P29 | Psychological sympt/compl other | 1 | 2 | 10 | 35 | 48 |
| N29 | Neurological sympt/complt other | 1 | 8 | 13 | 25 | 47 |
| W10 | Contraception postcoital | 0 | 0 | 0 | 46 | 46 |
| R02 | Shortness of breath/dyspnoea | 8 | 20 | 9 | 6 | 43 |
| -59 | Other therap proced/minor surg NEC | 12 | 14 | 9 | 7 | 42 |
| H99 | Ear/mastoid disease other | 8 | 15 | 13 | 6 | 42 |
| L01 | Neck symptom/complaint | 2 | 17 | 15 | 8 | 42 |
| A11 | Chest pain NOS | 0 | 2 | 10 | 29 | 41 |
| D99 | Disease digestive system other | 18 | 11 | 9 | 3 | 41 |
| N07 | Convulsion/seizure | 9 | 15 | 10 | 5 | 39 |
| S19 | Skin injury other | 2 | 12 | 14 | 10 | 38 |
| A08 | Swelling | 8 | 14 | 6 | 9 | 37 |
| S14 | Burn/scald | 11 | 8 | 7 | 10 | 36 |
| K01 | Heart pain | 0 | 1 | 9 | 23 | 33 |
| S70 | Herpes zoster | 0 | 5 | 17 | 10 | 32 |
| U70 | Pyelonephritis/pyelitis | 0 | 2 | 9 | 21 | 32 |
| A29 | General symptom/complaint other | 11 | 10 | 5 | 5 | 31 |
| S94 | Ingrowing nail | 1 | 1 | 7 | 22 | 31 |
| X99 | Genital disease female other | 2 | 4 | 4 | 19 | 29 |
| S74 | Dermatophytosis | 5 | 10 | 8 | 5 | 28 |
| R75 | Sinusitis acute/chronic | 0 | 6 | 4 | 17 | 27 |
| L15 | Knee symptom/complaint | 0 | 3 | 8 | 15 | 26 |
| T11 | Dehydration | 7 | 14 | 3 | 1 | 25 |
| L79 | Sprain/strain of joint NOS | 1 | 2 | 8 | 13 | 24 |
| S16 | Bruise/contusion | 6 | 5 | 10 | 3 | 24 |
| S18 | Laceration/cut | 2 | 6 | 8 | 8 | 24 |
| U06 | Haematuria | 2 | 7 | 9 | 6 | 24 |
| H81 | Excessive ear wax | 1 | 10 | 6 | 6 | 23 |
| R97 | Allergic rhinitis | 2 | 3 | 7 | 11 | 23 |
| X01 | Genital pain female | 0 | 8 | 8 | 6 | 22 |
| D09 | Nausea | 2 | 3 | 8 | 8 | 21 |
| L19 | Muscle symptom/complaint NOS | 0 | 0 | 8 | 13 | 21 |
| N89 | Migraine | 0 | 0 | 2 | 19 | 21 |
| U29 | Urinary symptom/complaint other | 2 | 11 | 4 | 4 | 21 |
| A87 | Complication of medical treatment | 5 | 4 | 5 | 6 | 20 |
| S13 | Animal/human bite | 1 | 9 | 7 | 3 | 20 |
| S29 | Skin symptom/complaint other | 5 | 4 | 4 | 7 | 20 |
| -30 | Medical examin/health eval complete | 7 | 3 | 5 | 4 | 19 |
| X* | Could be: X02 X05 X06 X07 | 4 | 0 | 2 | 13 | 19 |
| D88 | Appendicitis | 0 | 0 | 6 | 12 | 18 |
| A01 | Pain general/multiple sites | 1 | 4 | 7 | 5 | 17 |
| L13 | Hip symptom/complaint | 2 | 3 | 8 | 4 | 17 |
| N17 | Vertigo/dizziness | 0 | 1 | 4 | 12 | 17 |
| D71 | Mumps | 0 | 9 | 5 | 2 | 16 |
| P74 | Anxiety disorder/anxiety state | 0 | 2 | 1 | 13 | 16 |
| D95 | Anal fissure/perianal abscess | 4 | 3 | 4 | 4 | 15 |
| H04 | Ear discharge | 3 | 4 | 7 | 1 | 15 |
| R81 | Pneumonia | 0 | 9 | 4 | 2 | 15 |
| S02 | Pruritus | 0 | 9 | 4 | 2 | 15 |
| W29 | Pregnancy symptom/complaint other | 1 | 0 | 0 | 14 | 15 |
| D14 | Haematemesis/vomiting blood | 2 | 6 | 4 | 1 | 13 |
| D87 | Stomach function disorder | 1 | 2 | 2 | 8 | 13 |
| D89 | Inguinal hernia | 5 | 6 | 2 | 0 | 13 |
| D91 | Abdominal hernia other | 10 | 1 | 1 | 1 | 13 |
| S71 | Herpes simplex | 0 | 5 | 6 | 2 | 13 |
| X84 | Vaginitis/vulvitis NOS | 1 | 7 | 3 | 2 | 13 |
| D07 | Dyspepsia/indigestion | 0 | 0 | 3 | 9 | 12 |
| T90 | Diabetes non-insulin dependent | 0 | 0 | 4 | 8 | 12 |
| X06 | Menstruation excessive | 0 | 0 | 0 | 12 | 12 |
| F05 | Visual disturbance other | 0 | 1 | 4 | 6 | 11 |
| ** | "Foreign Body in Orifice" Could be: D79 H76 R87 -59 | 0 | 3 | 5 | 3 | 11 |
| H72 | Serous otitis media | 1 | 6 | 3 | 1 | 11 |
| N99 | Neurological disease other | 0 | 4 | 2 | 5 | 11 |
| T89 | Diabetes insulin dependent | 0 | 2 | 3 | 6 | 11 |
| R01 | Pain respiratory system | 1 | 0 | 3 | 6 | 10 |
| A04 | Weakness/tiredness general | 4 | 1 | 3 | 1 | 9 |
| L75 | Fracture: femur | 0 | 1 | 7 | 1 | 9 |
| P02 | Acute stress reaction | 0 | 0 | 0 | 9 | 9 |
| S05 | Lumps/swellings generalized | 1 | 3 | 1 | 4 | 9 |
| X* | Could be: X18 X19 X20 X21 X22 | 4 | 0 | 1 | 4 | 9 |
| -61 | Result examination/test/record/letter from other provider | 1 | 1 | 2 | 4 | 8 |
| B70 | Lymphadenitis acute | 1 | 2 | 4 | 1 | 8 |
| F79 | Injury eye other | 1 | 4 | 3 | 0 | 8 |
| L08 | Shoulder symptom/complaint | 0 | 2 | 1 | 5 | 8 |
| P01 | Feeling anxious/nervous/tense | 3 | 0 | 2 | 3 | 8 |
| P06 | Sleep disturbance | 3 | 2 | 1 | 2 | 8 |
| L76 | Fracture: other | 0 | 3 | 2 | 2 | 7 |
| P03 | Feeling depressed | 0 | 0 | 0 | 7 | 7 |
| P76 | Depressive disorder | 0 | 0 | 0 | 7 | 7 |
| S15 | Foreign body in skin | 0 | 5 | 2 | 0 | 7 |
| W78 | Pregnancy | 0 | 0 | 0 | 7 | 7 |
| Y74 | Orchitis/epididymitis | 0 | 0 | 1 | 6 | 7 |
| -31 | Medical examin/health eval partial | 3 | 1 | 1 | 1 | 6 |
| D84 | Oesophagus disease | 5 | 0 | 0 | 1 | 6 |
| F16 | Eyelid symptom/complaint | 3 | 2 | 1 | 0 | 6 |
| H76 | Foreign body in ear | 0 | 2 | 2 | 2 | 6 |
| L10 | Elbow symptom/complaint | 0 | 6 | 0 | 0 | 6 |
| S95 | Molluscum contagiosum | 0 | 3 | 3 | 0 | 6 |
| X02 | Menstrual pain | 0 | 0 | 0 | 6 | 6 |
| -66 | Referral to non-MD provider | 2 | 2 | 0 | 1 | 5 |
| A84 | Poisoning by medical agent | 1 | 3 | 0 | 1 | 5 |
| D06 | Abdominal pain localized other | 0 | 0 | 0 | 5 | 5 |
| N88 | Epilepsy | 1 | 0 | 1 | 3 | 5 |
| P99 | Psychological disorders other | 0 | 1 | 0 | 4 | 5 |
| R24 | Haemoptysis | 0 | 1 | 0 | 4 | 5 |
| R82 | Pleurisy/pleural effusion | 0 | 0 | 1 | 4 | 5 |
| U08 | Urinary retention | 0 | 4 | 1 | 0 | 5 |
| W14 | Contraception female other | 0 | 0 | 0 | 5 | 5 |
| X21 | Breast sympt/compl female other | 0 | 0 | 1 | 4 | 5 |
| Y04 | Penis symptom/complaint other | 2 | 0 | 3 | 0 | 5 |
| Y86 | Hydrocoele | 1 | 4 | 0 | 0 | 5 |
| Z25 | Assault/harmful event problem | 0 | 1 | 1 | 3 | 5 |
| -51 | I&d/flush/aspiration | 1 | 1 | 2 | 0 | 4 |
| A88 | Adverse effect physical factor | 0 | 4 | 0 | 0 | 4 |
| H73 | Eustachian salpingitis | 0 | 1 | 0 | 3 | 4 |
| K04 | Palpitations/awareness of heart | 0 | 0 | 1 | 3 | 4 |
| K29 | Cardiovascular sympt/complt other | 2 | 1 | 0 | 1 | 4 |
| L88 | Rheumatoid/seropositive arthritis | 0 | 0 | 2 | 2 | 4 |
| N91 | Facial paralysis/bells palsy | 0 | 0 | 2 | 2 | 4 |
| p71 | Organic psychosis other | 0 | 1 | 2 | 1 | 4 |
| S72 | Scabies/other acariasis | 1 | 1 | 0 | 2 | 4 |
| S85 | Pilonidal cyst/fistula | 0 | 0 | 0 | 4 | 4 |
| X90 | Genital herpes female | 0 | 0 | 0 | 4 | 4 |
| -44 | Preventive immunization/medic | 3 | 0 | 0 | 0 | 3 |
| A75 | Infectious mononucleosis | 0 | 0 | 0 | 3 | 3 |
| F76 | Foreign body in eye | 0 | 2 | 1 | 0 | 3 |
| K81 | Heart/arterial murmur NOS | 1 | 1 | 0 | 1 | 3 |
| K96 | Haemorrhoids | 0 | 1 | 0 | 2 | 3 |
| L80 | Dislocation/subluxation | 0 | 0 | 1 | 2 | 3 |
| N95 | Tension headache | 0 | 0 | 0 | 3 | 3 |
| S22 | Nail symptom/complaint | 0 | 2 | 0 | 1 | 3 |
| T87 | Hypoglycaemia | 1 | 1 | 0 | 1 | 3 |
| T99 | Endocr/metab/nutrit disease other | 0 | 1 | 0 | 2 | 3 |
| U04 | Incontinence urine | 0 | 1 | 1 | 1 | 3 |
| X08 | Intermenstrual bleeding | 0 | 0 | 0 | 3 | 3 |
| -60 | Result test/procedure | 0 | 0 | 1 | 1 | 2 |
| -43 | Other diagnostic procedure | 1 | 1 | 0 | 0 | 2 |
| -35 | Urine test | 0 | 2 | 0 | 0 | 2 |
| A71 | Measles | 1 | 1 | 0 | 0 | 2 |
| A73 | Malaria | 0 | 2 | 0 | 0 | 2 |
| A74 | Rubella | 1 | 0 | 1 | 0 | 2 |
| A86 | Toxic effect non-medicinal substance | 0 | 2 | 0 | 0 | 2 |
| D80 | Injury digestive system other | 0 | 0 | 1 | 1 | 2 |
| F75 | Contusion/haemorrhage eye | 0 | 0 | 1 | 1 | 2 |
| K99 | Cardiovascular disease other | 0 | 1 | 0 | 1 | 2 |
| L01 | Neck symptom/complaint | 0 | 0 | 2 | 0 | 2 |
| L86 | Back syndrome with radiating pain | 0 | 0 | 0 | 2 | 2 |
| L87 | Bursitis/tendinitis/synovitis NOS | 0 | 0 | 0 | 2 | 2 |
| R29 | Respiratory symptom/complaint other | 0 | 1 | 1 | 0 | 2 |
| S91 | Psoriasis | 0 | 1 | 0 | 1 | 2 |
| U95 | Urinary calculus | 0 | 0 | 0 | 2 | 2 |
| W80 | Ectopic pregnancy | 0 | 0 | 0 | 2 | 2 |
| X29 | Genital sympt/compl female other | 0 | 1 | 0 | 1 | 2 |
| Z29 | Social problem NOS | 0 | 1 | 1 | 0 | 2 |
| -68 | Other referral NEC | 1 | 0 | 0 | 0 | 1 |
| -58 | Therapeutic counselling/listening | 0 | 0 | 0 | 1 | 1 |
| -49 | Other preventive procedure | 0 | 1 | 0 | 0 | 1 |
| -42 | Electrical tracing | 0 | 0 | 1 | 0 | 1 |
| -34 | Blood test | 0 | 0 | 0 | 1 | 1 |
| -33 | Microbiological/immunological test | 0 | 0 | 0 | 1 | 1 |
| A91 | Abnormal result investigation NOS | 0 | 0 | 0 | 1 | 1 |
| A96 | Death | 0 | 0 | 1 | 0 | 1 |
| B80 | Iron deficiency anaemia | 0 | 0 | 0 | 1 | 1 |
| B82 | Anaemia other/unspecified | 0 | 0 | 1 | 0 | 1 |
| D13 | Jaundice | 1 | 0 | 0 | 0 | 1 |
| D15 | Melaena | 0 | 1 | 0 | 0 | 1 |
| D94 | Chronic enteritis/ulcerative colitis | 0 | 0 | 0 | 1 | 1 |
| H03 | Tinnitus, ringing/buzzing ear | 0 | 0 | 0 | 1 | 1 |
| H82 | Vertiginous syndrome | 0 | 0 | 0 | 1 | 1 |
| H86 | Deafness | 0 | 0 | 0 | 1 | 1 |
| K80 | Cardiac arrhythmia NOS | 0 | 0 | 1 | 0 | 1 |
| N06 | Sensation disturbance other | 0 | 0 | 0 | 1 | 1 |
| P15 | Chronic alcohol abuse | 0 | 0 | 0 | 1 | 1 |
| P72 | Schizophrenia | 0 | 0 | 0 | 1 | 1 |
| P80 | Personality disorder | 0 | 0 | 0 | 1 | 1 |
| P98 | Psychosis NOS/other | 0 | 0 | 0 | 1 | 1 |
| R86 | Benign neoplasm respiratory | 0 | 0 | 1 | 0 | 1 |
| W11 | Contraception oral | 0 | 0 | 0 | 1 | 1 |
| W82 | Abortion spontaneous | 0 | 0 | 0 | 1 | 1 |
| W92 | Compl labour/ delivery livebirth | 0 | 0 | 0 | 1 | 1 |
| Y71 | Gonorrhoea male | 0 | 0 | 1 | 0 | 1 |
| X* | Chlamydia infection genital female | 1 | 0 | 0 | 0 | 1 |
